# Electronic‐Structure‐Guided Screening of Piperazine Derivatives: Achieving Efficient CO_2_ Capture with High Cyclic Capacity and Low Energy Consumption

**DOI:** 10.1002/advs.202513855

**Published:** 2025-09-26

**Authors:** Feng Xie, Xuehua Shen, Han Lin, Junyang Lu, Feng Yan, Zuotai Zhang

**Affiliations:** ^1^ School of Environmental Science and Engineering Shenzhen Key Laboratory of Municipal Solid Waste Recycling Technology and Management Southern University of Science and Technology Shenzhen 518055 China; ^2^ Guangdong Provincial Key Laboratory of Soil and Groundwater Pollution Control Southern University of Science and Technology Shenzhen 518055 China

**Keywords:** CO_2_ capture, DFT‐based screening, piperazine derivatives, precipitation mechanism, solid‐liquid biphasic absorbents

## Abstract

Piperazine (PZ) is a promising alternative to monoethanolamine (MEA) for CO_2_ capture, yet its low desorption efficiency restricts practical implementation. Here, a density functional theory (DFT)‐driven framework is developed to identify PZ derivatives with improved performance. By integrating electronic descriptors—including molecular electrostatic potential (ESP) for absorption capacity, ESP at hydrogen nuclei (ESP_H) for desorption efficiency, activation barrier (ΔG‡) and Gibbs free energy change (ΔG) for absorption rate, and interaction‐region indicator (IRI) for energy consumption—the key performance of the derivatives is predicted. Experimental validation confirms the framework's predictive accuracy, with 2,6‐dimethylpiperazine (26DMPZ) identified as an optimal candidate. It demonstrates a desorption amount of 0.72 mol mol^−1^‐amine (≈80% higher than PZ), due to more negative ESP_H values. Furthermore, it shows 31% lower energy consumption compared to MEA, attributed to unstable dicarbamate formation. Spectroscopic analyses and solvation free energy calculations reveal that the low‐solubility of this dicarbamate salt further enhances CO_2_ capture performance without requiring organic solvents. Notably, the system retains 98.4% of its initial cyclic capacity over 10 rapid absorption–desorption cycles, indicating the industrial application potential. This study establishes an electronic‐structure‐guided screening strategy for amine solvents, highlighting precipitation‐driven phase separation as a tunable mechanism to optimize CO_2_ capture performance.

## Graphical abstract

A density functional theory (DFT)‐based framework is proposed to predict CO_2_ capture performance of piperazine derivatives. 2,6‐dimethylpiperazine (26DMPZ) is identified as a promising candidate: CO_2_‐induced precipitation in water eliminates the need for organic solvents; the low‐solubility, unstable dicarbamate enhances CO_2_ capture efficiency and reduces energy demand.

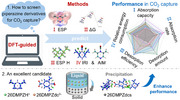

## 1 Introduction

In 2024, global CO_2_ emissions reached a record high of 41.6 billion tons,^[^ ^1^ ^]^ underscoring the limited effectiveness of current mitigation policies.^[^ ^2^, ^3^ ^]^ This surge in emissions has intensified the frequency of extreme weather events—such as wildfires, floods, and droughts—posing serious threats to human and ecological systems.^[^ ^4^ ^]^ To address this crisis, carbon capture, utilization, and storage (CCUS) technologies have emerged as pivotal strategies, with CO_2_ capture being the critical first step.^[^ ^5^, ^6^ ^]^ Among existing CO_2_ capture technologies, amine‐based chemical absorption is currently the most mature and industrially implemented,^[^ ^7^, ^8^ ^]^ with ongoing research focusing on achieving high absorption capacity, rapid absorption rates, large desorption amounts, and low regeneration energy consumption.^[^ ^9^ ^]^ Monoethanolamine (MEA)‐based aqueous solutions (typically 30 wt.%) have remained the benchmark in the industrial CO_2_ capture for years.^[^ ^10^ ^]^ Nonetheless, their application is constrained by several critical limitations, including poor thermal stability, low desorption efficiency (≈43.4% at 363.15 K),^[^ ^11^ ^]^ and high regeneration energy consumption (≈4.0 GJ/t‐CO_2_).^[^ ^12^ ^]^

To enhance the performance of MEA‐based CO_2_ capture systems, various modification strategies have been proposed, yet most still face notable limitations. For example, blended amine systems—such as diethanolamine (DEA)‐N‐methyldiethanolamine (MDEA) or MDEA‐MEA—leverage the components’ complementary properties to achieve a better balance between CO_2_ absorption rate and regeneration energy consumption.^[^ ^13^ ^]^ However, these mixtures often lead to reduced solvent stability, increased volatility, and greater uncertainty in corrosion behavior. The introduction of catalysts can accelerate reaction kinetics or reduce regeneration temperatures,^[^ ^14^ ^]^ but such formulations tend to be complex and may lead to solvent degradation or the formation of unknown byproducts during long‐term operation, increasing both operational and environmental risks. Biphasic solvent systems, proposed as a means to lower energy consumption, rely largely on organic solvents,^[^ ^15^ ^]^ which are associated with high volatility, elevated cost, and potential secondary pollution. In contrast, the development of MEA‐based structural derivatives, achieved by introducing functional groups to modulate the CO_2_ capture performance of the amine molecule, is currently one of the most promising approaches. This method allows for systematic, molecular‐level optimization of CO_2_ absorption capacity and thermal regeneration energy, while maintaining favorable environmental compatibility and industrial applicability. For example, Meng et al. explored the impact of different alkylation groups (e.g., methyl, butyl, ethyl) on CO_2_ absorption capacity and desorption efficiency,^[^ ^11^ ^]^ finding that ethylated MEA exhibited the most favorable regeneration characteristics. Another study has examined MEA derivatives incorporating varying hydroxyethyl groups, which showed improvements in regeneration characteristics.^[^ ^16^ ^]^ Furthermore, MEA derivatives with composite substitutions (e.g., methyl, butyl, ethyl, hydroxyethyl) have also been investigated, with certain derivatives demonstrating exceptional performance in CO_2_ absorption capacity and regeneration efficiency.^[^ ^17^ ^]^ The findings from these studies suggest that structural modifications to MEA can significantly improve the desorption efficiency and reduce regeneration energy consumption. However, due to the single amine group in each MEA molecule, its theoretical CO_2_ absorption capacity is capped at ≈0.5 mol mol^−1^‐amine,^[^ ^18^ ^]^ fundamentally limiting further performance improvements.

Consequently, piperazine (PZ), a cyclic diamine with two nitrogen atoms, offers both greater theoretical absorption capacity and higher thermal stability compared to MEA, making it a promising alternative. For example, previous studies have shown that an 8 M PZ aqueous solution can achieve a CO_2_ absorption capacity of 0.88 mol mol^−1^‐amine.^[^ ^19^ ^]^ Moreover, PZ remains thermally stable up to 423.15 K, outperforming widely conventional amines such as MEA, DEA, and MDEA.^[^ ^20^ ^]^ However, despite these advantages, PZ suffers from limited desorption efficiency, restricting its use primarily to absorption‐promoting roles. Previous studies have demonstrated that a 0.51 M PZ aqueous solution exhibits a cyclic CO_2_ capacity of 0.17 mol mol^−1^‐amine,^[^ ^21^ ^]^ with a notably low desorption efficiency of only 19.5%. These limitations greatly hinder PZ's potential as a primary amine in the development of advanced CO_2_ absorbent formulations. Inspired by the success of functional group optimization in MEA, a thorough investigation of PZ derivatives holds the potential to yield amines with enhanced desorption efficiency and absorption capacity. Some studies have investigated the impact of different alkylation substituents and functional groups on the CO_2_ absorption capacity and desorption efficiency of PZ derivatives,^[^ ^22^, ^23^ ^]^ suggesting that the influencing factors may be the electron donor effect and pK_a_, but the underlying mechanisms remain insufficiently explained. Moreover, existing amine screening strategies largely rely on fragmented empirical trials, wherein the mechanism driving the improvement of desorption efficiency remains inadequately elucidated, and it is uncertain whether pK_a_ alone governs this improvement.^[^ ^24^, ^25^ ^]^ This highlights the lack of a comprehensive mechanistic analysis of the performance differences among these derivatives and failed to conduct an exhaustive evaluation of the structure‐performance relationship.

In recent years, Density functional theory (DFT) calculations have increasingly been used in the study of amine‐based chemical absorption for CO_2_ capture.^[^ ^26^, ^27^ ^]^ For instance, Tang et al. analyzed the electronic properties of PZ derivatives to explain the effects of various substituents on CO_2_ absorption enthalpy (ΔH_abs_) and equilibrium solubility (α_e_), attributing the superior performance of triethylenediamine (TEDA) to steric hindrance effects.^[^ ^22^ ^]^ Jia et al. employed DFT to calculate Gibbs free energy for assessing the regeneration energy requirements and CO_2_ absorption kinetics of alkylated PZ derivatives.^[^ ^23^ ^]^ DFT was also applied to calculate dipole moments and intermolecular hydrogen bonding of amine─CO_2_ for exploring the phase change behavior.^[^ ^28^ ^]^ These studies demonstrate that DFT calculations have shown great potential in predicting the CO_2_ capture abilities of amines. However, current studies using DFT calculations mainly focus on explaining specific mechanisms,^[^ ^29^, ^30^ ^]^ and the theoretical results often differ from experimental data. This discrepancy primarily arises from two factors: (1) prior DFT investigations have typically been limited in scope and have failed to establish an integrated computational framework tailored to liquid amine systems that comprehensively addresses adsorption performance, desorption performance, and energy consumption; (2) solvent effects have often been overlooked, and standardized parameterization has not been systematically implemented, despite their critical role in accurately modeling amine performance in CO_2_ capture processes,^[^ ^31^ ^]^ particularly with respect to substituent‐dependent kinetic barriers. Therefore, establishing a robust and holistic analysis system based on DFT calculation is essential for understanding the relationship between structure and performance in liquid amine systems, and for reliably predicting the CO_2_ capture performance in novel liquid amine systems.

In this study, we established a comprehensive DFT‐based prediction framework to assess the CO_2_ capture performance of PZ derivatives. By analyzing molecular electrostatic potential (ESP) distributions, ESP at hydrogen nuclei (ESP_H), activation barrier (ΔG‡), and Gibbs free energy change (ΔG) for zwitterion formation, and interaction‐region indicators (IRI), we systematically predicted key performance metrics, including absorption capacity, absorption rate, desorption efficiency, and energy consumption. The theoretical predictions were further validated through bench‐scale experiments. Notably, in some cases, discrepancies between theoretical prediction and experimental data were observed due to unexpected precipitate formation. We addressed this through in‐depth mechanistic analysis using solution‐state ^13^C nuclear magnetic resonance (NMR) spectroscopy, high‐resolution mass spectrometry (HRMS), Fourier‐transform infrared (FTIR) spectroscopy, and solvation free energy (ΔG_solv_) calculations. This integrative approach not only refines our predictive model but also highlights precipitation as a previously underappreciated factor in CO_2_ capture design. Overall, this study delivers a validated theoretical strategy for predicting CO_2_ capture performance, enabling rational design of efficient amine‐based absorbents.

## 2 Results and Discussions

### 2.1 DFT‐Guided Screening Framework for PZ Derivatives

**Figure 1:**
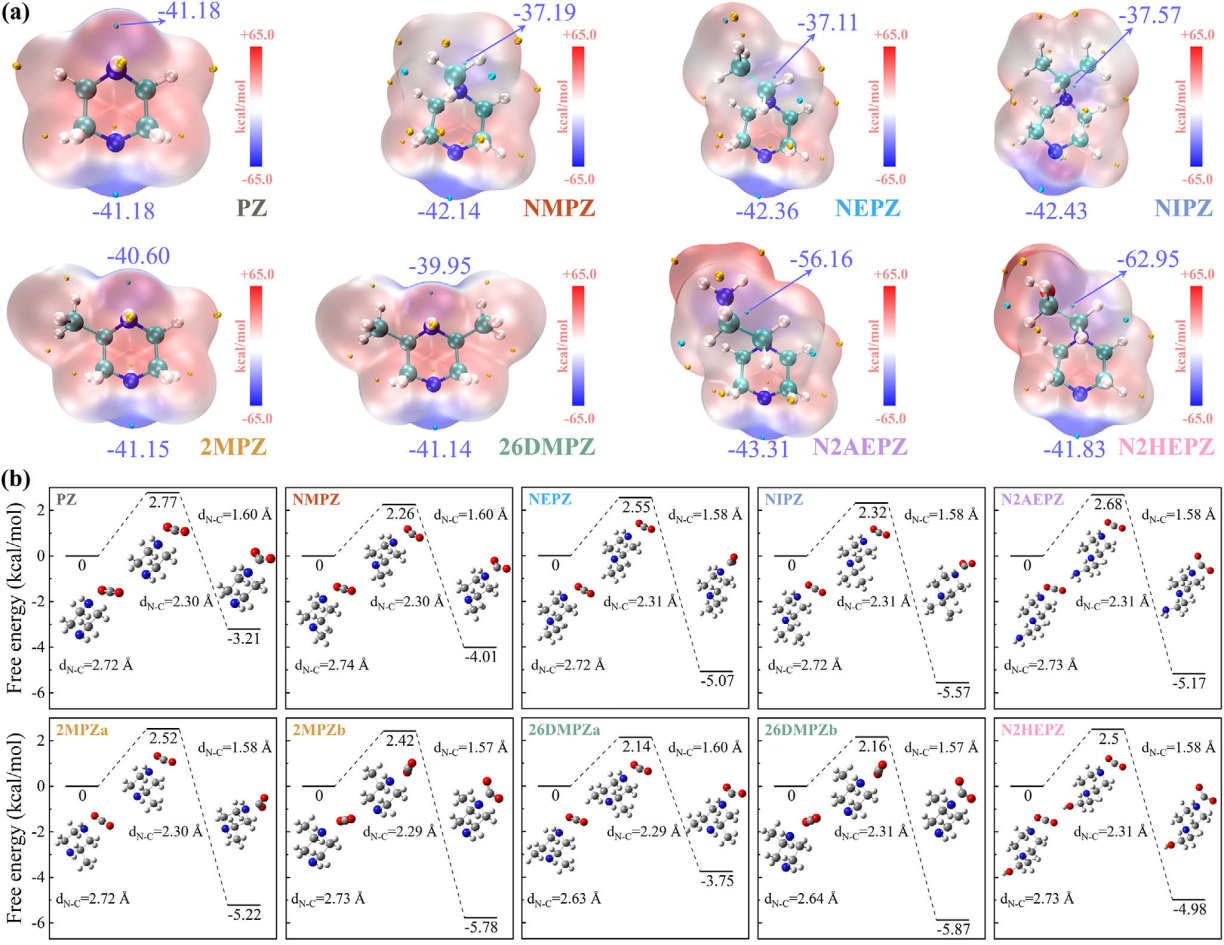
a) ESP maps of PZ derivatives and b) ΔG‡ and ΔG for the formation of zwitterion via the reaction between PZ derivatives and CO_2_.

While the pK_a_ of amines reflects nitrogen basicity,^[^ ^32^ ^]^ it does not fully capture their site‐specific reactivity toward CO_2_. To better assess absorption behavior, we analyzed the molecular ESP distributions of eight PZ derivatives, focusing on the ESP minima near each nitrogen atom (**Figure** **1**a). In unsubstituted PZ, the local ESP minima near the two nitrogen atoms are both −41.18 kcal mol^−1^, serving as a reference for comparison. For mono‐alkyl‐substituted derivatives—N‐methylpiperazine (NMPZ), N‐Ethylpiperazine (NEPZ), and N‐Isopropylpiperazine (NIPZ)—ESP analysis reveals that the alkyl group reduces the electron density near the substituted nitrogen (more positive ESP minima), while slightly increasing ESP minima negativity near the unsubstituted nitrogen atom. As the alkyl group grows in size, this effect becomes more pronounced. Importantly, once nitrogen becomes tertiary, it no longer participates directly in carbamate formation due to steric hindrance and instead functions as a proton acceptor.^[^ ^33^ ^]^ As a result, the predicted absorption capacity follows the order: NMPZ < NEPZ < NIPZ < PZ.

For adjacent methyl substitutions—2‐methylpiperazine (2MPZ) and 2,6‐dimethylpiperazine (26DMPZ)—ESP minima near both nitrogen atoms decrease as the number of methyl groups increases, reflecting reduced nucleophilicity and thus lower reactivity toward CO_2_. This trend suggests decreasing absorption capacity in the order: 26DMPZ < 2MPZ < PZ. In the aminoethyl‐substituted derivative—N‐(2‐Aminoethyl)piperazine (N2AEPZ)—both nitrogen sites show obviously more negative ESP minima, with the substituted site reaching −56.16 kcal mol^−1^. This indicates enhanced CO_2_ reactivity, potentially due to the presence of a third amine group.^[^ ^34^ ^]^ Among all derivatives, N2AEPZ is predicted to have the highest absorption capacity. For N‐(2‐Hydroxyethyl)piperazine (N2HEPZ), although the ESP minimum near the substituted nitrogen is highly negative (due to the hydroxyl group),^[^ ^35^ ^]^ the opposite site shows a less favorable ESP minimum than that in NEPZ, implying overall lower reactivity. As a result, its predicted absorption capacity is lower than that of NEPZ.

The absorption rates of primary and secondary amines are primarily governed by the kinetics of carbamate formation.^[^ ^36^ ^]^ Among the elementary steps, zwitterion formation is typically regarded as the rate‐determining step.^[^ ^27^ ^]^ To assess the kinetics of this step, we computed the ΔG‡ and ΔG for zwitterion formation (Equation 1) between each secondary amine and CO_2_ (Figure 1b). For the mono‐alkyl‐substituted derivatives NMPZ, NEPZ, and NIPZ, ΔG becomes progressively more negative compared to unsubstituted PZ, indicating enhanced thermodynamic favorability for zwitterion formation. Concurrently, the ΔG‡ show a slight decreasing trend: 2.26 kcal mol^−1^ for NMPZ, 2.32 kcal mol^−1^ for NIPZ, and 2.77 kcal mol^−1^ for PZ. Although the differences in activation energies are relatively modest, the ΔG changes are more substantial and better reflect reactivity. NIPZ, with the most negative ΔG (−5.57 kcal mol^−1^), is thus predicted to have the highest absorption rate among the four. The resulting order of predicted CO_2_ absorption rates is: NIPZ > NEPZ > NMPZ > PZ.

(1) RNH+CO2→RNH+COO−

Methyl substitution at positions adjacent to the secondary amine nitrogen introduces electronic asymmetry, giving rise to two distinct nitrogen environments. Accordingly, for 2MPZ and 26DMPZ, we computed ΔG‡ and ΔG at both the nitrogen distal to the methyl group and the one adjacent to it. In 2MPZ, the distal nitrogen exhibits a lower ΔG‡ and more negative ΔG compared to the adjacent site, suggesting that CO_2_ preferentially reacts at the distal position. A similar pattern is observed in 26DMPZ, where the ΔG for the distal site is −5.87 kcal mol^−1^, substantially more negative than the −3.75 kcal mol^−1^ observed at the adjacent site. These findings suggest that the dominant reaction pathway proceeds via the nitrogen farther from the methyl substituent. As a result, the predicted absorption rate trend for these three molecules is: 26DMPZ > 2MPZ > PZ. For N2AEPZ and N2HEPZ, both ΔG‡ and ΔG are comparable to those of NEPZ. These results suggest that their kinetic behavior in CO_2_ absorption is similar, and their rates are likely to be on par with NEPZ. The relative energy trends were confirmed to be robust through high‐level validation: 1) using larger basis sets (def2‐TZVPP and def2‐QZVP) at the M06‐2X‐D3(0) level, and 2) employing the double‐hybrid revDSD‐PBEP86‐D3(BJ) functional with the def2‐TZVPP basis set (Tables S1 and S2, Supporting Information). This agreement affirms the reliability of our M06‐2X‐D3(0)/def2‐TZVP approach for cost‐effective screening.

While zwitterion formation governs the kinetics of CO_2_ absorption, the regeneration capability of amines is primarily determined by the thermodynamics and kinetics of carbamate decomposition during desorption.^[^ ^37^ ^]^ For primary and secondary amines, this process relies on the proton returning to the amine nitrogen and the subsequent detachment of CO_2_ (Equation 2). We hypothesize that the strength of electrostatic attraction between the amine and the proton plays a critical role in facilitating carbamate decomposition. To assess this, we evaluated the ESP_H in the amine group as a proxy for desorption efficiency. For obtaining a physically meaningful ESP value at the position of the hydrogen nucleus, the self‐interaction of the nucleus was excluded from the calculation, thereby avoiding artificially infinite electrostatic potentials. A more negative ESP_H implies stronger attraction and greater likelihood of proton reassociation, thereby favoring desorption.

(2) RNCOO−+H+→RNH+CO2

**Figure 2:**
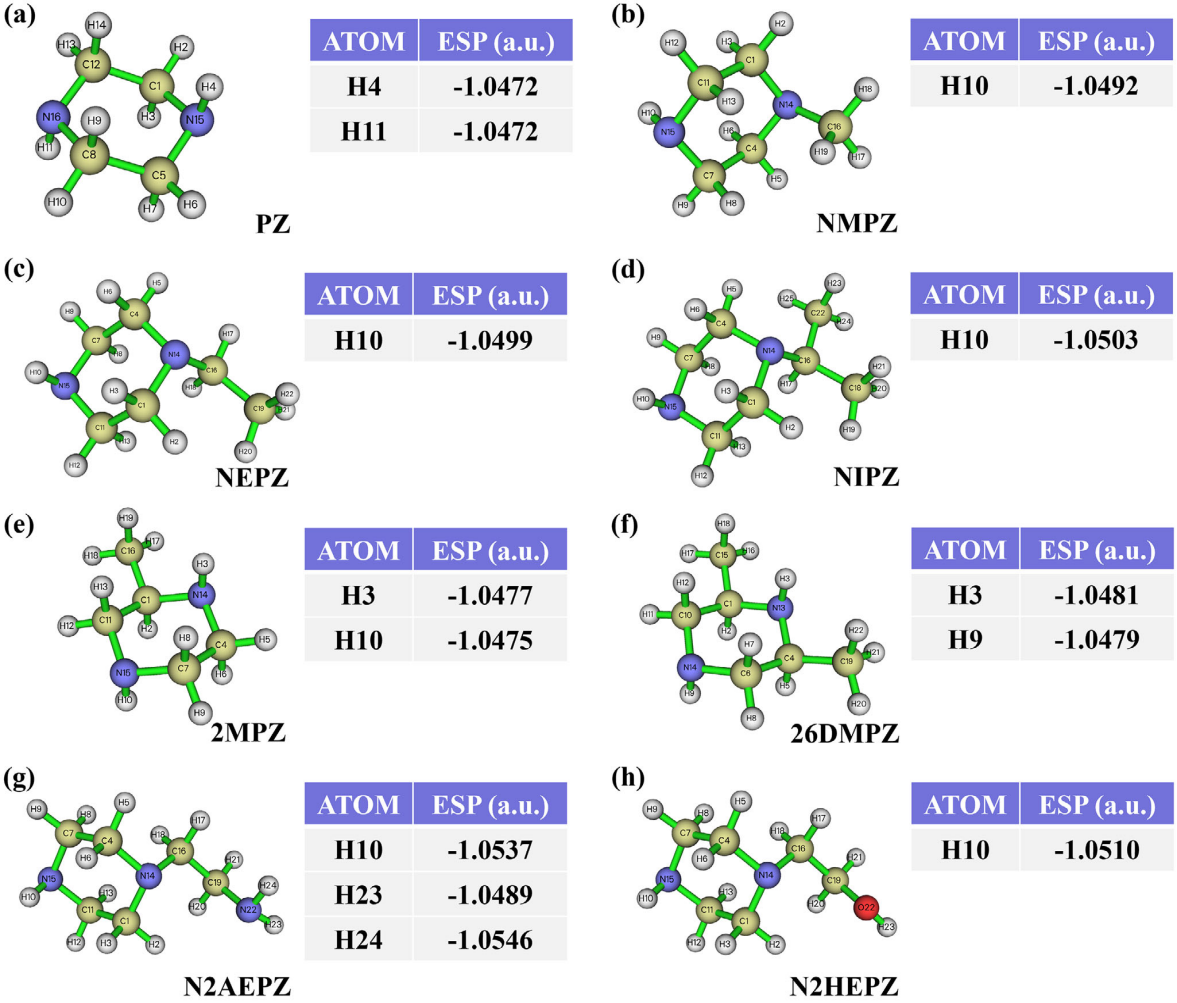
The ESP_H values of amino groups in PZ derivatives: reflecting the strength of proton affinity and the potential for enhanced desorption performance across different derivatives.

ESP_H values for all PZ derivatives were computed using Multiwfn 3.8, and the results are summarized in **Figure** **2**. In unsubstituted PZ (Figure 2a), both secondary amine groups show identical ESP_H values of −1.0472 a.u., owing to the molecule's symmetric structure. Upon alkyl substitution at one nitrogen atom (e.g., methyl, ethyl, or isopropyl groups), the ESP_H of the opposite amine becomes progressively more negative. This observation suggests that bulkier substituents enhance the electron‐donating capacity of the derivatives, which could strengthen electrostatic interactions with the proton, thereby improving CO_2_ desorption efficiency. As shown in Figure 2e,f, adjacent methyl substitution leads to a marked increase in ESP_H negativity. 2MPZ exhibits ESP_H values of −1.0477 and −1.0475 a.u. at its two secondary amine sites, while 26DMPZ further decreases these to −1.0481 and −1.0479 a.u. This consistent downward trend implies that increasing the number of methyl groups amplifies electron donation and thus strengthens proton binding, potentially enhancing CO_2_ desorption efficiency. In N2AEPZ (Figure 2g), the ethyl group in NEPZ is replaced by an amino group, introducing a primary amine. As a result, the ESP_H of the remaining secondary amine becomes more negative (−1.0537 a.u.), indicating enhanced desorption potential. However, the presence of a primary amine introduces two additional protons (H_23_ and H_24_), which also display relatively negative ESP_H values. Since primary amines can lose both protons during desorption, the resulting protonation dynamics are more complex, potentially reducing desorption efficiency despite favorable ESP_H values. Similarly, in N2HEPZ (Figure 2f), the ethyl group is replaced with a hydroxyethyl substituent. The hydroxyl group contributes electron density, resulting in a more negative ESP_H at the secondary amine, comparable to that observed in N2AEPZ. This shift suggests that N2HEPZ may exhibit slightly enhanced desorption performance relative to NEPZ.

**Figure 3:**
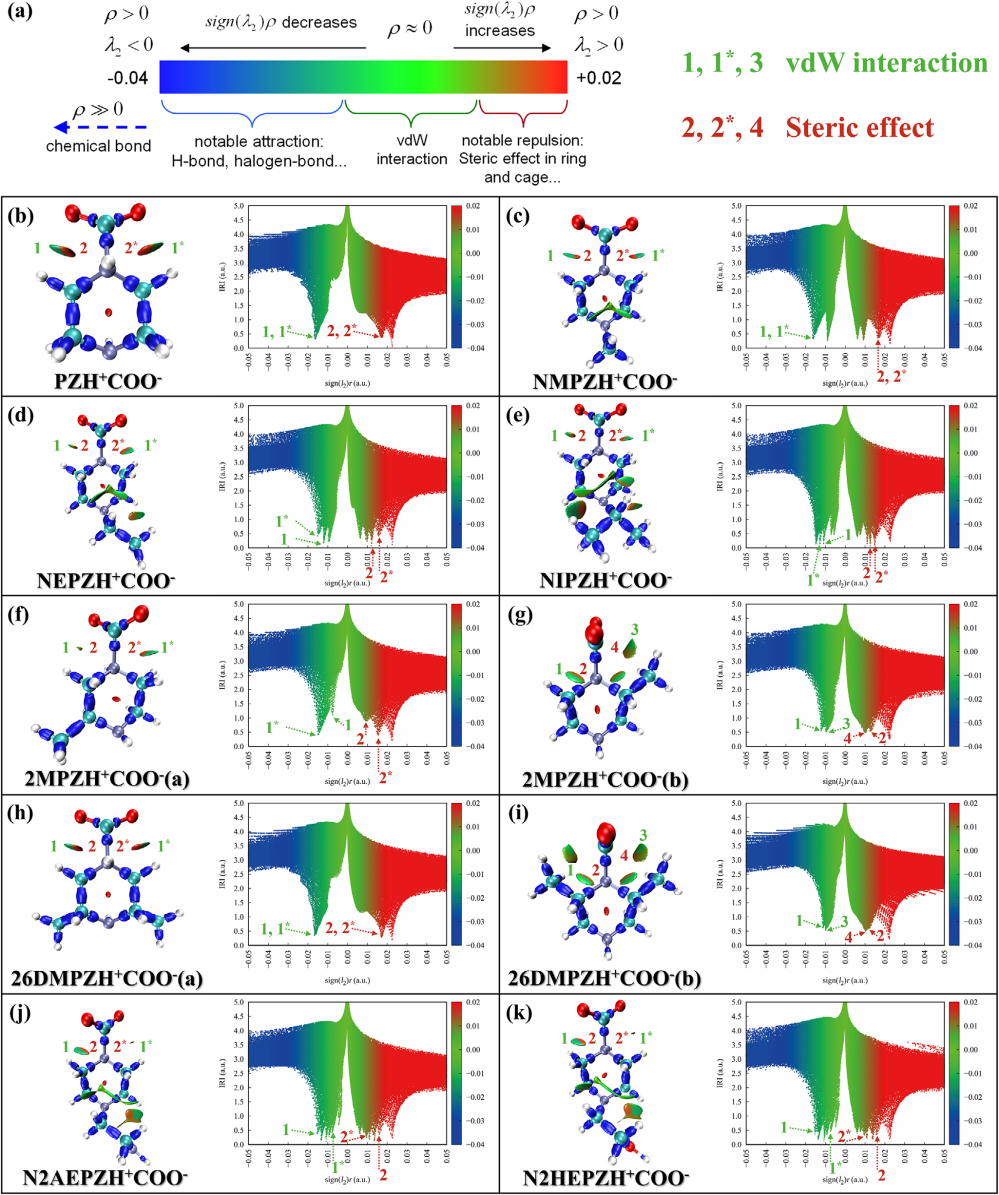
IRI isosurface maps of zwitterion structures in PZ derivatives and corresponding reduced density gradient (RDG) vs sign (λ_2_)ρ scatter plots: insights into steric and electrostatic interactions.

Steric hindrance is known to influence the energy cost of carbamate decomposition during CO_2_ desorption.^[^ ^38^, ^39^ ^]^ To explore this effect across PZ derivatives, we employed IRI and AIM (Atoms‐In‐Molecules) analysis to evaluate weak intermolecular interactions and quantify the N─C bond strength in zwitterionic intermediates. **Figure** **3** presents the IRI isosurface maps and sign(λ_2_)ρ scatter plots for each derivative. IRI surfaces are color‐coded to differentiate interaction types: green regions indicate van der Waals (vdW) interactions, while red regions reflect steric repulsion. For unsubstituted PZ (Figure 3b), both steric and vdW interactions are symmetrically distributed around the two amine‐CO_2_ interaction sites, as evidenced by corresponding peaks in the scatter plots. Alkyl substitution at the nitrogen atom modifies this interaction landscape. In NMPZ, NEPZ, and NIPZ (Figure 3c–e), the steric repulsion slightly decreases compared to PZ, while internal vdW interactions emerge within the amine backbone. This shift implies that alkyl substitution alleviates steric hindrance, enhancing the effective interaction between the amine and CO_2_. AIM analysis (Figure S1, Supporting Information) supports this interpretation: the N─C bond strength increases across the series PZ < NMPZ < NEPZ < NIPZ, indicating more stable carbamate formation. These results collectively suggest higher regeneration energy demand for larger alkyl substituents.

For adjacent methyl substitutions (2MPZ and 26DMPZ), two reaction pathways were considered: one involving the nitrogen distal to the methyl group, and one involving the adjacent nitrogen. In 2MPZ (Figure 3f,g), condition (a) shows reduced steric hindrance, while condition (b) introduces additional vdW interactions and moderate steric effects. A similar pattern is observed for 26DMPZ. In 26DMPZ (Figure 3h,i), condition (a) resembles PZ, while condition (b) closely mirrors 2MPZ. AIM analysis further reveals that the N─C bond is strengthened in both pathways for 2MPZ compared to PZ, whereas in 26DMPZ, condition a is slightly weaker than PZ, but condition b shows comparable bonding. Based on these findings, the predicted energy consumption trend is: PZ < 26DMPZ < 2MPZ.

For heteroatom‐functionalized derivatives (N2AEPZ and N2HEPZ), the IRI maps (Figure 3j,k) reveal weaker steric interactions between the amine and CO_2_ relative to NEPZ, likely due to increased flexibility and polarity.^[^ ^40^ ^]^ Also, AIM analysis shows stronger N─C bonds than in NEPZ, indicating more stable carbamate formation. Together, these results suggest that both reduced steric repulsion and strengthened N─C bonding contribute to more stable carbamate formation, resulting in higher energy consumption.

Based on the above theoretical calculations and analyses, a DFT‐based predictive framework was developed to systematically assess the CO_2_ capture performance of PZ derivatives. The framework incorporates ESP distributions, ESP_H, ΔG‡, ΔG for zwitterion formation, and IRI to predict key performance metrics, including absorption capacity, absorption rate, desorption efficiency, and energy consumption. Through this prediction framework, 26DMPZ was predicted as the optimal absorbent among eight PZ derivatives, exhibiting relatively low energy consumption, alongside a high absorption rate and desorption efficiency. Although its absorption capacity is slightly lower than that of PZ, its overall performance outperforms the other derivatives. This predictive framework may provide valuable theoretical insights for the design of efficient amine‐based absorbents and serve as a powerful tool for screening new CO_2_ capture materials.

### 2.2 Experimental Validation of CO_2_ Capture Performance

**Figure 4:**
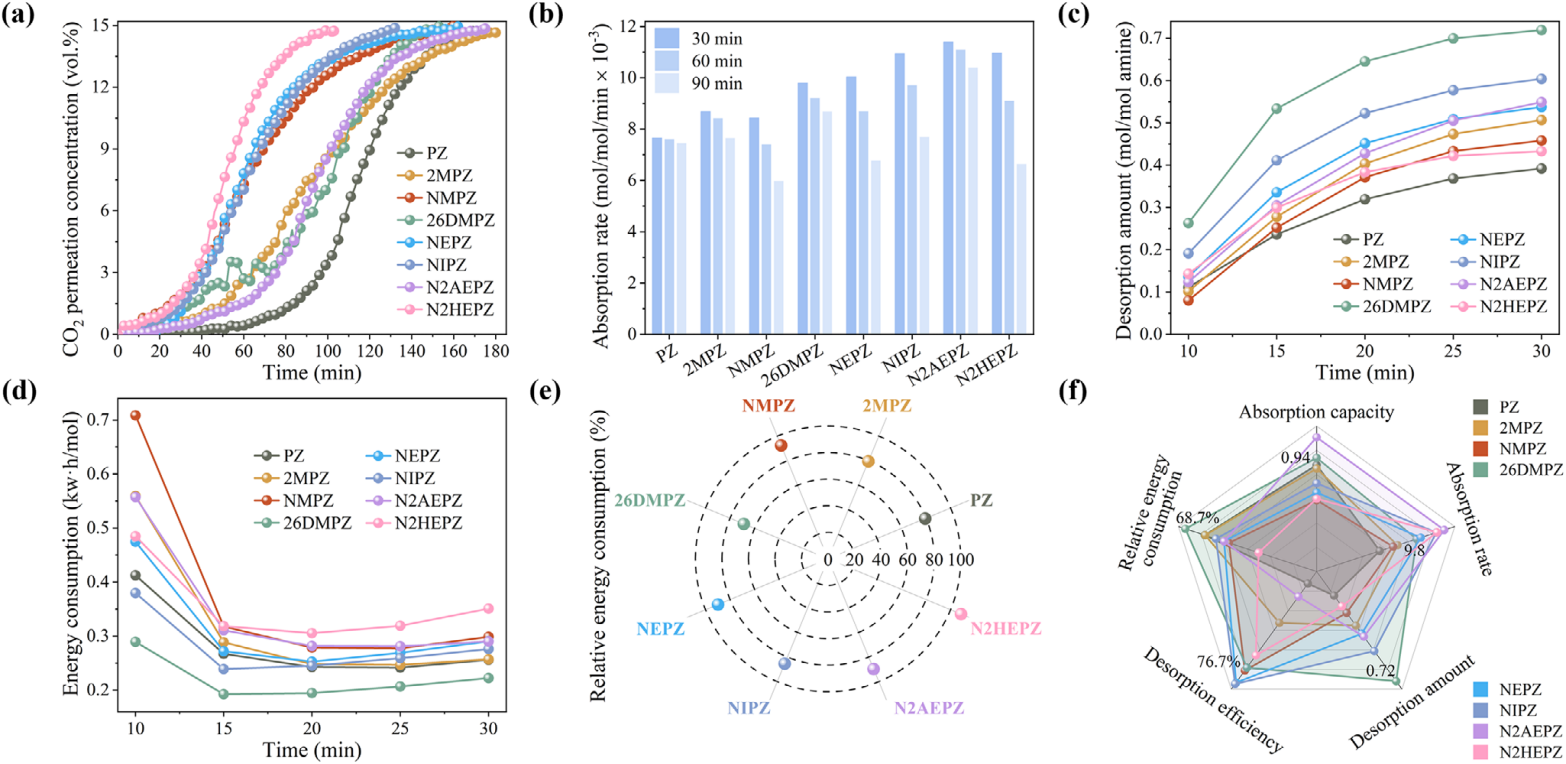
Experimental evaluation of CO_2_ absorption and desorption performance of PZ derivatives: a) CO_2_ breakthrough concentration during the absorption process; b) average CO_2_ absorption rates at 30, 60, and 90 min; c) variation in CO_2_ desorption amount over 30 min; d) time‐dependent energy consumption during desorption; e) relative energy consumption compared to 30 wt.% MEA; f) comprehensive performance comparison of each derivative.

In this section, the experimental results are compared with the theoretical predictions presented in Section 2.1. The goal is to assess the reliability of the computational framework and identify factors influencing CO_2_ capture performance among PZ derivatives. Key performance parameters such as absorption capacity, absorption rate, desorption capacity, and energy consumption have been tested (**Figure** **4**). The breakthrough curves used to determine CO_2_ absorption capacities are shown in Figure 4a. Among all tested amines, PZ displays the highest absorption capacity at 0.88 mol mol^−1^‐amine, with the derivatives ranked as follows: NMPZ (0.59) < N2HEPZ (0.60) < NEPZ (0.65) < NIPZ (0.73) < 2MPZ (0.85) < PZ (0.88) < 26DMPZ (0.94) < N2AEPZ (1.11). For N‐alkyl‐substituted derivatives, the experimental results are consistent with predictions: alkylation converts secondary to tertiary amines, reducing reactivity toward CO_2_.^[^ ^41^ ^]^ 2MPZ, with a single methyl on the adjacent carbon, shows slightly reduced absorption capacity compared to PZ, matching theoretical expectations. 26DMPZ, however, exceeds expectations with 0.94 mol mol^−1^‐amine, potentially due to precipitate formation, which—per Le Chatelier's principle—drives the equilibrium toward carbamate formation.^[^ ^42^ ^]^ This hypothesis is strongly supported by a control experiment using a 10 wt.% 26DMPZ solution, in which no precipitation was observed (Figure S7a, Supporting Information). The absorption capacity of this homogeneous solution was 0.85 mol mol^−1^‐amine, which is in excellent agreement with our initial DFT‐based prediction and provides a critical baseline. The enhancement to 0.94 mol mol^−1^‐amine achieved in the precipitating 30 wt.% system offers direct experimental evidence that the formation of a poorly soluble solid product is the key factor enhancing the ultimate CO_2_ absorption capacity. N2AEPZ achieves the highest absorption capacity, consistent with the presence of multiple amine groups. N2HEPZ exhibits lower absorption capacity than NEPZ, again aligning with ESP‐based predictions. These results are consistent with the ESP distribution predictions, where derivatives with more negative ESP minima near nitrogen atoms typically display higher absorption capacities.

To further assess the absorption rate, the average CO_2_ absorption rates at 30, 60, and 90 min were calculated, as shown in Figure 4b. In general, compounds with more favorable zwitterion formation thermodynamics (lower ΔG‡ and more negative ΔG) show faster absorption. The observed trends agree with theoretical predictions, except for N2AEPZ, which absorbs CO_2_ more rapidly than expected. This discrepancy is explained by the primary amine in N2AEPZ, whose zwitterion formation thermodynamics are comparable to secondary amines in PZ (Figure S2, Supporting Information). Furthermore, the molecule's structural diversity—containing primary, secondary, and tertiary amines—enhances its capacity to act as a multi‐site proton acceptor,^[^ ^43^ ^]^ boosting absorption kinetics.

Next, Figure 4c shows the variation in CO_2_ desorption amounts over time for each derivative. After 30 min, all amines have almost completed desorption. Despite PZ's high absorption, it shows the lowest desorption amount (0.39 mol mol^−1^‐amine) and efficiency (44.6%). For N‐alkyl‐substituted amines, desorption efficiency increases with substituent size: PZ (44.6%) < NMPZ (77.8%) < NEPZ (82.5%) < NIPZ (83.0%). For adjacent‐substituted derivatives, the trend is also consistent with predictions: PZ (44.6%) < 2MPZ (59.6%) < 26DMPZ (76.7%). N2AEPZ shows a moderate desorption amount (0.55 mol mol^−1^‐amine), lower than NEPZ despite higher absorption, again consistent with prediction. N2HEPZ shows lower‐than‐expected efficiency, possibly due to hydroxyl groups stabilizing carbamates and impeding CO_2_ release.^[^ ^44^ ^]^ These results validate that more negative ESP_H values lead to better desorption efficiency, as predicted by the theoretical model.

Finally, the time‐dependent energy consumption data, along with the energy consumption values relative to 30 wt.% MEA, are presented in Figure 4d,e. PZ's energy consumption is 79.2% of MEA. For N‐alkyl‐substituted derivatives, energy consumption increases with substituent size, as predicted. For 2MPZ, energy use is slightly above PZ, matching theory. 26DMPZ, however, exhibits the lowest energy consumption (68.7% of MEA)—contrary to predictions. We emphasize that this reduction is specific to our laboratory bench‐scale apparatus and desorption operation. Its core value is to provide a fast and reliable relative metric for preliminary screening. N2AEPZ also shows unexpectedly low energy consumption, possibly due to the energetically favorable behavior of its primary amine group. For N2HEPZ, the desorption energy consumption is in line with the prediction, being indeed higher than NEPZ, but the energy consumption is excessively high, which can be attributed to the influence of the hydroxyl group.^[^ ^44^ ^]^ The general trend confirms the findings from the IRI and AIM analyses: weaker steric repulsion between the amine and CO_2_, together with a weaker N─C bond, both contribute to reducing energy consumption.

Overall, the experimental results for CO_2_ absorption and desorption across most PZ derivatives corroborate the theoretical predictions presented earlier. Key performance indicators—including absorption capacity, absorption rate, desorption efficiency, and energy consumption—show strong consistency with the computational model, validating the reliability of the prediction framework. Notably, among all derivatives, 26DMPZ exhibited the best overall CO_2_ capture performance (Figure 4f). Its absorption rate and desorption efficiency aligned well with theoretical expectations. Furthermore, its absorption capacity in a non‐precipitating, homogeneous system (0.85 mol mol^−1^‐amine, 10 wt.%) closely matched our DFT‐based prediction, confirming the model's accuracy for the solution‐phase reaction. The enhanced absorption capacity (0.94 mol mol^−1^‐amine) observed at higher concentration is therefore unequivocally attributed to the occurrence of precipitation, which shifts the reaction equilibrium via Le Chatelier's principle. Besides, the unexpectedly low desorption energy consumption may also be related to the formation of the precipitate. Therefore, understanding the mechanism of precipitate formation and its thermodynamic properties is crucial not only to explain the observed deviations in 26DMPZ's performance but also to provide a more comprehensive interpretation of the model's predictive outcomes.

### 2.3 Mechanistic Investigation of Precipitation‐Enhanced CO_2_ Capture

To elucidate the mechanism underlying the unexpected performance enhancement of 26DMPZ—particularly its higher absorption capacity (promoted by precipitation) and lower regeneration energy—we investigated the formation and properties of precipitates during CO_2_ absorption through comprehensive characterization of the solid products and further DFT calculations.

**Figure 5:**
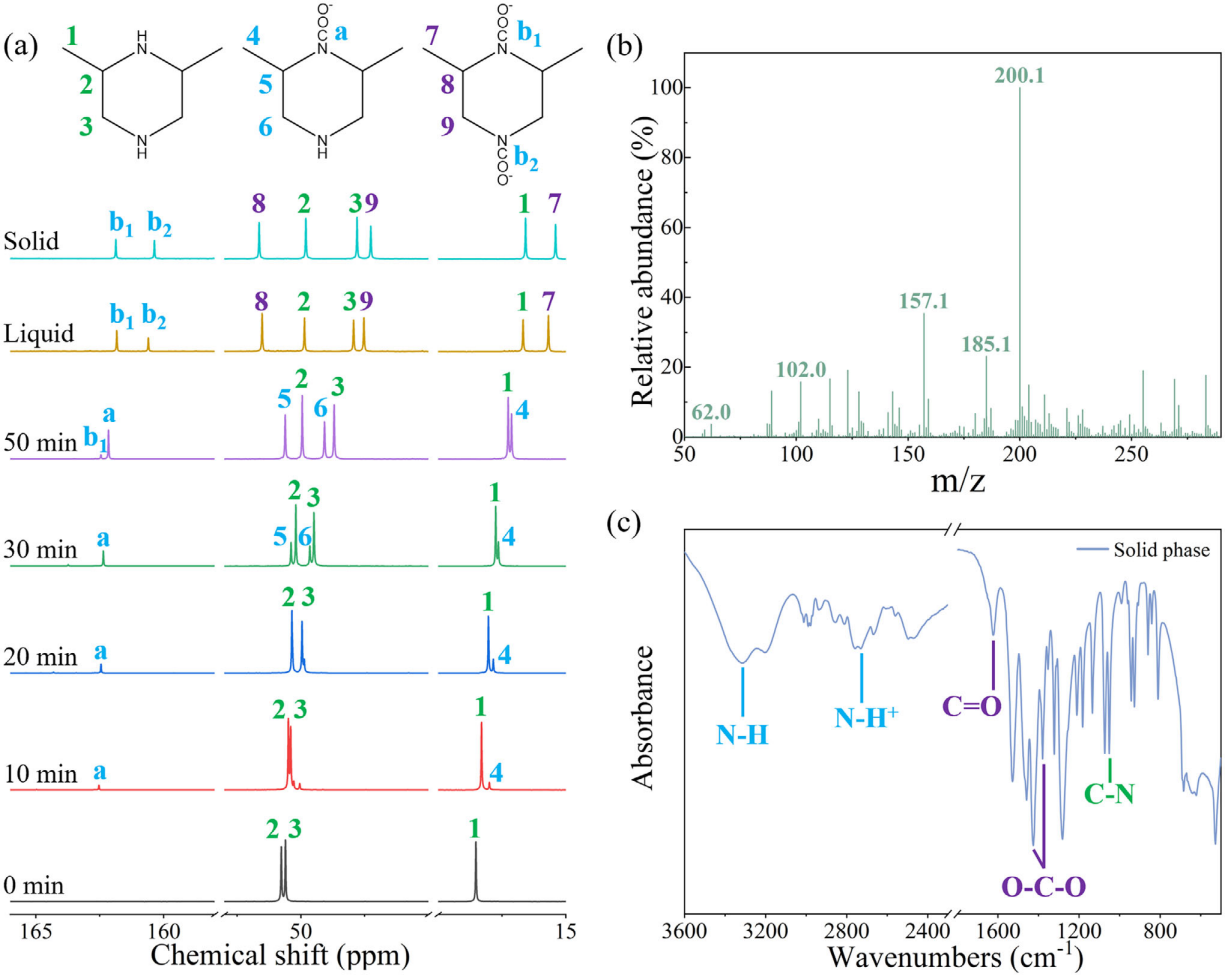
a) ^13^C NMR spectra of 26DMPZ during the CO_2_ absorption process; b) negative‐mode electrospray ionization (ESI) mass spectrum of the solid product; c) FTIR spectrum of the solid product.

As shown in **Figure** **5**a, the ^13^C NMR spectra clearly illustrate the structural evolution of 26DMPZ upon exposure to CO_2_. At the initial stage (0 min), only three sharp peaks were observed—two around ≈50.6 ppm attributed to ring carbons and one at ≈18.5 ppm corresponding to a methyl carbon—indicating the presence of unreacted 26DMPZ. Upon CO_2_ introduction (10 and 20 min), a new peak appeared at ≈161.8 ppm in the carboxyl region, suggesting the formation of a mono‐substituted carbamate intermediate.^[^ ^31^ ^]^ By 30 min, additional ring carbon peaks (C_5_, C_6_) emerged, while the carboxyl region continued to display a dominant single peak, indicating that mono‐substituted species remained the primary product. At 50 min, a second carboxyl signal (≈162.3 ppm) became evident; shortly thereafter, the solution turned turbid, implying that disubstituted carbamate formation occurred in tandem with precipitation. In the final solid product isolated from the reaction, two distinct carboxyl resonances (≈161.8 and ≈160.4 ppm) were detected, clearly confirming the presence of a disubstituted carbamate species.

The HRMS analysis (Figure 5b) further supports this structural assignment. A dominant ion peak at m/z = 200.10 matches the calculated mass of the deprotonated disubstituted carbamate anion, confirming its identity as the major product. A secondary peak at m/z = 157.10 is attributable either to the mono‐substituted species or to a fragment generated from the disubstituted structure during ionization. Additionally, a low‐intensity peak at m/z = 61.98 corresponds to bicarbonate (HCO_3_^−^), consistent with minor side reactions. Since bicarbonate decomposes at a significantly lower temperature than carbamate and is present in negligible amounts (undetectable by NMR), it does not impact the system's cyclic capacity. The cationic mass spectrum (Figure S3, Supporting Information) reveals a significant peak at m/z = 115, attributed to the protonated amine species of unreacted 26DMPZ, further supporting the presence of free amine in its protonated form. FTIR analysis (Figure 5c) provides complementary evidence for the formation of carbamate species in the precipitate. A strong absorption band at 1680 cm^−1^ corresponds to the C═O stretching vibration of the carbamate group.^[^ ^45^ ^]^ Additional symmetric O─C─O stretching bands were observed at 1458 and 1425 cm^−1^.^[^ ^46^ ^]^ Broad N─H stretching bands centered around ≈3300 cm^−1^ (neutral N─H) and ≈3100–2800 cm^−1^ (protonated N─H^+^) indicate the presence of both hydrogen bonding and protonated amine functionalities.^[^ ^47^ ^]^ The characteristic C─N stretching band near 1100 cm^−1^ further confirms that both nitrogen atoms on the PZ ring underwent substitution.^[^ ^48^ ^]^ Taken together, these combined spectroscopic results conclusively demonstrate that the observed precipitate is primarily composed of disubstituted carbamate products formed from 26DMPZ upon reaction with CO_2_.

Since the dicarbamate species is the dominant component in both the liquid and solid phases, we performed further DFT calculations and thermogravimetric analysis coupled with differential scanning calorimetry (TGA‐DSC) to understand its decomposition behavior. DFT calculations on the reaction pathway show that the formation of the dicarbamate intermediate from the monocarbamate has a ΔG‡ of 3.82 kcal mol^−1^ and ΔG of 1.67 kcal mol^−1^ (Figure S4, Supporting Information). In contrast, the ΔG‡ and ΔG for the formation of the initial monocarbamate are 2.16 and −5.87 kcal mol^−1^, respectively. The positive ΔG and higher ΔG‡ for the second carbamation step indicate that the dicarbamate is intrinsically less stable and more prone to decomposition than the monocarbamate, which is the fundamental molecular‐level reason for its low regeneration energy requirement. Moreover, this computational prediction was confirmed experimentally by TGA‐DSC analysis. The solid product began to decompose rapidly at ≈60 °C, with ≈50% mass loss achieved by 100 °C (Figure S5, Supporting Information). Two distinct endothermic peaks at ≈92.9 and ≈112.3 °C coincide with the mass‐loss steps, indicating the low‐energy, stepwise release of CO_2_. The complete decomposition of the solid product occurs below 120 °C, which is significantly lower than the decomposition temperatures reported for solid products in other solid‐liquid biphasic systems,^[^ ^49^, ^50^, ^51^ ^]^ suggesting a further energy‐saving advantage if only the solid phase is regenerated in future process designs.

Furthermore, to evaluate the practical handleability and reproducibility of the precipitation process, the particle size distribution (PSD) of the dried solid was determined by laser diffraction. The results (Table S3 and Figure S6, Supporting Information) show a consistent PSD with a median particle size (D50) of 83.7 ± 13.9 µm and D90 of 186.3 ± 8.6 µm, confirming the formation of filterable, micrometer‐sized particles. The low standard deviations of D50 and D90 across repeated measurements demonstrate excellent reproducibility of the precipitation process, which is an essential prerequisite for reliable industrial operation. Breakthrough experiments (Figure S7, Supporting Information) conducted in reactors of different diameters demonstrated that the precipitation onset is governed by the absorbed CO_2_ loading rather than hydrodynamic artifacts, supporting the robustness of this mechanism across operational scales.

**Figure 6:**
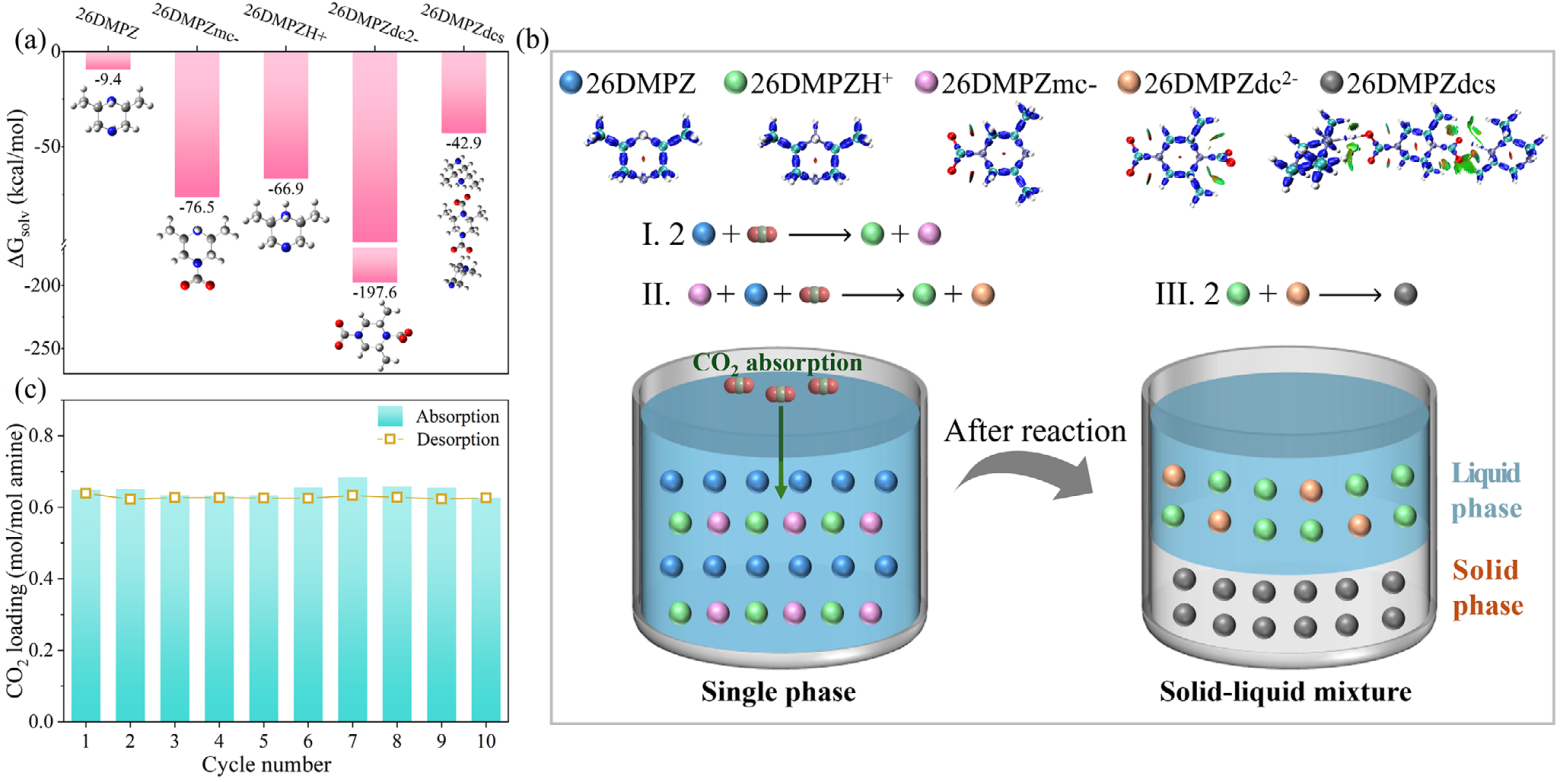
a) ΔG_solv_ of key species involved in the reaction of CO_2_ absorption with 26DMPZ; b) proposed mechanism for CO_2_ absorption and precipitation pathway; c) cyclic performance of 26DMPZ under rapid absorption‐desorption conditions.

To further elucidate the thermodynamic driving force behind the precipitation observed during the CO_2_ absorption process with 26DMPZ, we performed DFT calculations of ΔG_solv_ for key species involved in the reaction, as shown in **Figure** **6**a. Unreacted 26DMPZ exhibits a moderately negative ΔG_solv_ (−9.4 kcal mol^−1^), while the mono‐substituted carbamate anion (26DMPZmc^−^) and the protonated form (26DMPZH^+^) display more negative values (−76.5 and −66.9 kcal mol^−1^, respectively), indicating enhanced aqueous solubility. Notably, the dicarbamate dianion (26DMPZdc^2−^) possesses an extremely negative ΔG_solv_ (−197.6 kcal mol^−1^), suggesting a high degree of stability and solubility in water. However, upon combination with two equivalents of 26DMPZH^+^, the resulting neutral dicarbamate salt (26DMPZdcs) shows an obviously less negative ΔG_solv_ of −42.9 kcal mol^−1^. Although still negative, this represents a substantial reduction in solvation tendency compared to the highly soluble ionic precursors. In other words, the transformation from highly soluble ionic species to a poorly soluble neutral salt constitutes the thermodynamic basis for precipitation.

Figure 6b illustrates the proposed mechanism. Initially, two molecules of 26DMPZ react with CO_2_ to form carbamate intermediates. These intermediates then undergo further reaction with CO_2_ to yield the dicarbamate dianion. Finally, the dianion associates with two protonated 26DMPZH^+^ molecules via ionic interactions, forming a low‐solubility dicarbamate salt, which induces a phase transition from a homogeneous liquid to a biphasic solid–liquid system, resulting in precipitation. To further investigate the cyclic performance of 26DMPZ, we first analyzed the regenerated solution using NMR spectroscopy (Figure S8, Supporting Information). The results indicated no detectable degradation products, with the principal component being intact 26DMPZ molecules along with a small amount of persistent monocarbamate, demonstrating good chemical stability of the amine solvent under these regeneration conditions. Furthermore, based on desorption amount data (Figure 4c), 90.3% of the maximum desorption amount (0.65 mol mol^−1^‐amine) was achieved at 20 min. Therefore, we conducted ten consecutive absorption–desorption cycles using a 20‐min desorption time. As shown in Figure 6c, in the first cycle, the CO_2_ absorption capacity was 0.65 mol mol^−1^‐amine with a desorption amount of 0.64 mol mol^−1^‐amine; by the tenth cycle, the absorption capacity was 0.63 mol mol^−1^‐amine and the desorption amount was 0.63 mol mol^−1^‐amine. Thus, after ten cycles, the desorption capacity remained at 98.4% of the initial value, demonstrating competitive performance among comparable absorbents and highlighting outstanding cyclic stability.^[^ ^29^, ^49^ ^]^

### 2.4 Environmental Implications and Performance Benchmarking

This study establishes a DFT‐guided predictive framework for screening amine‐based CO_2_ absorbents, utilizing a range of electronic‐structure‐based descriptors. ESP analysis near the nitrogen atoms was used to predict absorption capacity, with more negative ESP values indicating higher absorption capacity. ΔG‡ and ΔG for zwitterion formation were used to predict absorption rate, with more negative ΔG and lower ΔG‡ corresponding to faster absorption rates. ESP_H served as a proxy for proton affinity, with more negative ESP_H values indicating better desorption efficiency. Additionally, IRI and AIM analysis were used to evaluate steric interactions and bond strength, with stronger N─C bonds and reduced steric hindrance indicating higher energy consumption during desorption. Compared to traditional experimental screening methods, which are often costly and time‐consuming, this theoretical approach is modular, resource‐efficient, and easily scalable to large compound libraries. With predictive accuracy validated by bench‐scale experiments, this method enables the rapid and rational identification of high‐performance absorbents at the molecular design stage, accelerating the discovery of sustainable CO_2_ capture materials.

Through the application of the DFT‐guided predictive framework, we identified 26DMPZ as the most promising CO_2_ absorbent. To clearly position the performance of 26DMPZ among state‐of‐the‐art liquid amine absorbents, it is instructive to categorize them based on their composition and phase‐change behavior: 1) homogeneous aqueous absorbents, e.g., conventional 30 wt.% MEA;^[^ ^11^, ^17^ ^]^ 2) organic solvent‐dependent biphasic absorbents, which require solvents like NMP or DMSO to induce phase separation;^[^ ^29^, ^30^, ^51^, ^53^ ^]^ and 3) organic solvent‐free aqueous biphasic absorbents, a nascent class where phase separation occurs via solid precipitation in water without organic additives. Our 26DMPZ/H_2_O system is an exemplar of this third, most sustainable category. As benchmarked in **Table** **1**, 26DMPZ delivers an absorption capacity of 0.94 mol mol^−1^‐amine and a desorption amount of 0.72 mol mol^−1^‐amine, both of which are highly competitive compared to other piperazine derivatives in the current literature. Crucially, and in stark contrast to organic solvent‐dependent systems, 26DMPZ achieves this via an organic solvent‐free precipitation mechanism. This fundamental advantage is quantified by its exceptional desorption amount of 42.4 L kg^−1^‐absorbent, which out‐performs systems that appear attractive on a molar basis but operate at much lower amine concentrations. Specifically, Traditional solid–liquid biphasic absorbents often rely on organic solvents to achieve phase separation.^[^ ^55^ ^]^ However, the solubility of amines in organic solvents is limited, and higher amine concentrations can adversely affect absorption performance. As a result, many of these systems operate at relatively low amine concentrations, which reduces their absolute absorption capacity. Furthermore, while these systems may appear to have high desorption capacities when measured in mol/mol‐amine, their desorption efficiency (measured in terms of L/kg‐ or L‐absorbent) is often relatively low due to the dilution effects of the solvent and the limited solubility of amines at higher concentrations. In contrast, 26DMPZ functions in water at a high effective amine concentration without sacrificing kinetics, giving it a clear edge in both absorption capacity and desorption efficiency. Its performance is further underpinned by the low regeneration energy of the solid product, as confirmed by TGA‐DSC analysis showing complete decomposition below 120 °C.

**Table 1:** Comparison of the CO_2_ capture performance of the PZ derivatives in this study with other reported absorbents.

Absorbents	Organic solvent requirement	Concentration	Absorption amount	Desorption amount		Refs.
			[mol/mol‐amine]	[mol/mol‐amine]	[L/kg‐absorbent]	
MEA/H_2_O^a)^	No	30 wt.%	0.59	0.26	28.2	[17]
DMMEA/H_2_O^a)^	No	3 M	0.62	0.41	27.7^e)^	[11]
MMEA/MDEA/H_2_O^a)^	No	4.9 M	0.62	0.34	36.8^e)^	[17]
AMP/PZ/H_2_O^a)^	No	4.9 M	0.57	0.30	32.4^e)^	[52]
AEEA/DMSO/PMDETA^b)^	Yes	1 M	1.75	1.18	26.4^e)^	[53]
TETA/AMP/NMP^b)^	Yes	1 mol kg^−1^	0.94	0.77	17.2	[29]
PD/PZ/NMP^b)^	Yes	1 mol kg^−1^	0.86	0.78	17.5	[30]
AMP/PZ/DME^b)^	Yes	1 mol kg^−1^	0.87	0.82	18.4	[49]
PZ/DMF^b)^	Yes	0.2 M	0.82	N.A.^d)^	N.A.^d)^	[50]
AEP/DMSO/H_2_O^b)^	Yes	≈20 wt.%	1.05	N.A.^d)^	N.A.^d)^	[51]
PZ/AMP/NMF^b)^	Yes	≈1.3 M	1.20	1.12	≈32.6	[54]
**26DMPZ/H_2_O** ^c)^	**No**	30 wt.%	0.94	0.72	**42.4**	**This work**

^a)^ homogeneous aqueous absorbents;

^b)^ organic solvent‐dependent biphasic absorbents;

^c)^ organic solvent‐free aqueous biphasic absorbents;

^d)^ not available;

^e)^ L/L‐absorbent.

Abbreviations: DMMEA, dimethyl monoethanolamine; MMEA, methyl monoethanolamine; AMP, 2‐amino‐2‐methyl‐1‐propanol; AEEA, 2‐((2‐aminoethyl)amino)ethanol; DMSO, dimethyl sulfoxide; PMDETA, pentamethyldiethylenetriamine; TETA, triethylenetetramine; NMP, N‐methyl‐2‐pyrrolidone; PD, 4‐amino‐1‐methylpiperidine; DME, dipropylene glycol dimethyl ether; DMF, N,N‐dimethylformamide; AEP, N‐aminoethylpiperazine; NMF, N‐methylformamide.

Beyond CO_2_ capture performance metrics, 26DMPZ offers distinct sustainability advantages. Because it operates in an all‐aqueous medium, it eliminates the organic solvents required by conventional biphasic formulations such as PD/PZ/NMP or PZ/DMF.^[^ ^30^, ^50^ ^]^ This avoids solvent‐related volatility, toxicity, and waste‐handling issues, while sidestepping the concentration‐performance compromises those systems face. The absence of volatile organic compounds (VOCs) lowers environmental risk, simplifies plant integration, and reduces solvent make‐up costs. Besides, we screened corrosivity by Tafel polarization on carbon steel (Figure S9, Supporting Information): Under the same conditions, when there was no CO_2_ loading, the corrosion currents (I_corr_) of 26DMPZ and MEA solutions were 8.00 × 10^−7^ A and 1.65 × 10^−6^ A, respectively; the I_corr_ of the CO_2_‐loaded 26DMPZ solution was 7.32×10^−6^ A, which was lower than 1.60×10^−5^ A of CO_2_‐loaded MEA; the results showed that the 26DMPZ absorbent also had advantages over MEA in terms of corrosion resistance. Additionally, supplementary tests at 5 vol.% CO_2_ (Figure S10, Supporting Information) showed an absorption capacity of 0.89 mol mol^−1^‐amine and a desorption amount of 0.68 mol mol^−1^‐amine, confirming that 26DMPZ maintains good performance under lower CO_2_ partial pressure. Combined with its low energy demand for regeneration and the excellent stability (98.4% cyclic capacity retention over 10 cycles) demonstrated over ten rapid absorption–desorption cycles, 26DMPZ emerges as a green, scalable candidate for industrial CO_2_ capture. Leveraging its precipitation‐driven, water‐based phase separation, it exemplifies how electronic‐structure‐based predictions and process‐level sustainability can converge to advance next‐generation CCUS technologies.

## 3 Conclusion

In this study, we established a robust DFT‐guided framework for screening high‐performance PZ derivatives for CO_2_ capture. By integrating electronic descriptors—including ESP for absorption capacity, ESP_H for desorption efficiency, ΔG‡/ΔG for absorption kinetics, and IRI for steric interactions—we successfully predicted key performance metrics of eight PZ derivatives. Experimental validation confirmed the framework's accuracy, with 26DMPZ identified as the optimal candidate. Its exceptional performance stems from two synergistic effects:

1. Electronic modulation: Methyl substitution generates highly negative ESP_H values (−1.0481 a.u.), enhancing proton affinity and enabling 80% higher desorption capacity than PZ (0.72 vs. 0.39 mol mol^−1^‐amine).
2. Precipitation‐driven phase separation: The formation of a low‐solubility dicarbamate salt shifts reaction equilibrium (ΔG_solv_ = −42.9 kcal mol^−1^), boosting absorption capacity to 0.94 mol mol^−1^‐amine; the 31% lower regeneration energy vs MEA arises primarily from the dicarbamate's intrinsic lability.

Notably, the aqueous 26DMPZ system achieved 98.4% cyclic capacity retention over 10 rapid cycles, demonstrating industrial viability without organic solvents. This work not only provides an electronic‐structure–guided screening strategy for amine absorbents but also establishes transferable design principles—solubility and phase‐behavior control—for aqueous amine CO_2_ capture systems.

## 4 Experimental Section

### Chemicals

Mixed gas (15% CO_2_ and 85% N_2_) was supplied by Huatepeng Gas Co., Ltd. (Shenzhen, China). The PZ derivatives, including PZ (≥99%), N‐methylpiperazine (NMPZ, ≥99%), N‐ethylpiperazine (NEPZ, ≥98%), N‐isopropylpiperazine (NIPZ, ≥98%), 2‐Methylpiperazine (2MPZ, ≥98%), 2,6‐Dimethylpiperazine (26DMPZ, ≥98%), N‐(2‐Aminoethyl)piperazine (N2AEPZ, ≥98%), and N‐(2‐Hydroxyethyl)piperazine (N2HEPZ, ≥99%) were purchased from Shanghai Macklin Biochemical Co., Ltd. and used to prepare the solid–liquid biphasic absorbents. Deuterium oxide (D_2_O; ≥99.9%), obtained from Shanghai Aladdin Biochemical Technology Co., Ltd., was used in nuclear magnetic resonance (NMR) spectroscopy.

### Computational Methods

All quantum‐chemical calculations were carried out with Gaussian 16.^[^ ^56^ ^]^ Solvent effects were modeled using the SMD (solvation model based on solute electron density) continuum model in water,^[^ ^57^ ^]^ with London dispersion interactions included using Grimme's D3(0) correction method.^[^ ^58^ ^]^ This setup was well‐suited for high‐throughput screening and for capturing comparative reactivity trends across a molecular series, as shown in prior applications.^[^ ^22^, ^29^ ^]^ However, it is noted that recent work has demonstrated that explicit, dynamical solvent coupling can qualitatively alter CO_2_‐capture kinetics and decouple barrier heights from observed rates; accordingly, the barrier analysis was intended for relative ranking rather than ab initio rate prediction.^[^ ^59^ ^]^ Within this comparative framework, the reported barriers should be interpreted as screening metrics rather than absolute kinetics. First, the geometries of the eight PZ derivatives were optimized at the M06‐2X‐D3(0)/def2‐SVP level. The optimized structures were then analyzed for ESP,^[^ ^60^ ^]^ IRI,^[^ ^61^ ^]^ and AIM (Atoms‐In‐Molecules),^[^ ^62^ ^]^ using Multiwfn 3.8,^[^ ^63^, ^64^ ^]^ with visualizations produced in VMD software.^[^ ^65^ ^]^ Next, at the same M06‐2X‐D3(0)/def2‐SVP level, transition states for the reaction of each derivative with CO_2_ to form the zwitterionic intermediates were located, and intrinsic reaction coordinate (IRC) calculations were performed to verify their connections to the corresponding reactants and products. Finally, single‐point energy calculations on reactants, transition states, and products were conducted at the M06‐2X‐D3(0)/def2‐TZVP level, and Shermo 2.6 was used to apply harmonic‐frequency corrections and compute ΔG at 313.15 K.^[^ ^66^ ^]^

### Experimental Design and Characterization

All CO_2_ absorption and desorption experiments were carried out in a bench‐scale apparatus (Figure S11, Supporting Information). For absorption tests, a 15 vol% CO_2_/N_2_ mixture was first humidified by passing through a water saturator, then fed at 400 mL min^−1^ into a three‐neck flask containing 30 wt.% PZ derivative solution. The solution was maintained at 40 °C using an electric heating mantle (LICHEN SZCL‐250 mL, China), and the outlet CO_2_ concentration was monitored in real time by a flue‐gas analyzer (GAS Tiger 6000–2L, China) until breakthrough. The total CO_2_ uptake was calculated from the time‐resolved concentration profile, and the average absorption rate was determined from the cumulative uptake at 30, 60, and 90 min. For desorption, the CO_2_‐loaded solution was heated to 100 °C; the combined heating and isothermal hold lasted 30 min in total. The cumulative CO_2_ released was measured by a mass‐flow controller (Sevenstar‐CS200, China), and the corresponding electrical energy consumption was recorded via a power meter (UT230A‐II, China) connected to the heating mantle.^[^ ^67^ ^]^ To ensure like‐for‐like comparability across solvents, CO_2_‐loaded 26DMPZ solution was regenerated directly as a mixed solid–liquid slurry within the reaction apparatus. In cycling tests on 26DMPZ, the desorption time was shortened to 20 min; all other conditions remained identical. To elucidate the precipitation mechanism of 26DMPZ during the reaction, samples were collected at 0, 10, 20, 30, and 50 min during absorption, as well as both the liquid and solid phases after absorption, for a total of seven samples. All samples were analyzed by ^13^C NMR on a Bruker Ascend 400 MHz spectrometer (relaxation delay 20 s, 180 scans). The solid products were freeze‐dried to remove residual water prior to analysis. FT‐IR spectra of the dried solids were recorded over 400–4000 cm^−1^ (Thermo Fisher Nicolet iS50), and HRMS (Thermo Scientific QE Plus) was used to acquire both positive and negative ESI spectra. Finally, ΔG_solv_ of the zwitterionic and precipitated species were computed using DFT; full computational details are provided in the Supporting Information.

## Conflict of Interest

The authors declare no conflict of interest.

## Supporting information



Supporting Information

## Data Availability

The authors declare that the main data supporting the findings of this study are available within the article and its supporting information files. Extra data are available from the corresponding authors upon reasonable request.
