# Supplementary material for: Electronic‐Structure‐Guided Screening of Piperazine Derivatives: Achieving Efficient CO2 Capture with High Cyclic Capacity and Low Energy Consumption
Source: Adv Sci (Weinh). 2025 Sep 26;12(46):e13855. doi: 10.1002/advs.202513855 (PMC12697836; doi:10.1002/advs.202513855)
Supplement: Supplementary file 1 — Supporting Information [file ADVS-12-e13855-s001.docx]

Supporting Information for

**Theoretical screening of piperazine derivatives for CO_2_ capture: Unveiling the impact of precipitation on enhanced capture performance**

*Feng Xie,^a^ Xuehua Shen,^a,b,*^ Han Lin,^a^ Junyang Lu,^a^ Feng Yan,^a,b,c,^ Zuotai Zhang^a,b,c,*^*

^a^School of Environmental Science and Engineering, Shenzhen Key Laboratory of Municipal Solid Waste Recycling Technology and Management, Southern University of Science and Technology, Shenzhen 518055, China.

^b^Guangdong Provincial Key Laboratory of Soil and Groundwater Pollution Control, Southern University of Science and Technology, Shenzhen 518055, China.

^c^Guizhou Green Industry Technology Institute, Guiyang 550014, China.

**^*^ Corresponding author:**

E-mail address: [shenxh@sustech.edu.cn](mailto:shenxh@sustech.edu.cn); [zhangzt@sustech.edu.cn](mailto:zhangzt@sustech.edu.cn)

Number of Pages: 13

Number of Texts: 3

Number of Tables: 4

Number of Figures: 14

The Supporting Information file includes the following sections:

Supporting Texts S1-S3S2

Supporting Tables S1-S4S4

Supporting Figures S1-S14S7

**Text S1.** **Calculation of ΔG_solv_**

Solvation free energy (ΔG_solv_), also known as the free energy of solvation,^[1]^ is calculated as follows:

$$\text{Δ}\text{G}\text{solv}\text{=G}\text{sol}\text{n}-\text{G}\text{gas}$$

where G_solv_ is the single-point energy in the solution phase, calculated under an implicit solvent model (SMD), using the M05-2X/6-31G* level of theory; G_gas_ is the single-point energy in the gas phase, also calculated at the M05-2X/6-31G* level.

**Text S2. Detailed description of computational descriptor**

1) Electrostatic potential (ESP): The electrostatic potential, *V*(r), at a point r in space is defined by the interaction energy between the molecular charge distribution and a positive unit test charge placed at that point. In our study, the local minima of *V*(r) near nitrogen atoms directly represents their nucleophilicity. A more negative value indicates a stronger attraction between the nitrogen's lone pair and the electrophilic carbon atom of CO_2_, thus predicting a higher absorption capacity.

2) Electrostatic potential at hydrogen nuclei (ESP_H): The electrostatic potential at a nucleus would theoretically be infinite due to the self-interaction of the point charge. Therefore, to obtain a physically meaningful quantum mechanical descriptor of proton affinity, we calculate the electrostatic potential at the nucleus while excluding the contribution of its own nuclear charge. Therefore, the ESP_H is equivalent to the electrostatic interaction energy between its atomic nucleus (i.e., a proton) and the rest of the system. A more negative ESP_H implies stronger attraction and greater likelihood of proton reassociation (RNCOO^–^ + H^+^ → RNH + CO_2_), thereby favoring desorption.

3) Activation Barrier (ΔG‡) and Gibbs Free Energy Change (ΔG): The ΔG‡ represents the energy barrier between the reactants and the transition state and determines the kinetics of a chemical reaction. The ΔG reflects the thermodynamic driving force, with negative values indicating exergonic and spontaneous processes. In this study, ΔG‡ and ΔG for the zwitterion formation step (amine + CO_2_ → zwitterion) are used to predict the CO_2_ absorption rate of a series of PZ derivatives.

4) Atoms-in-Molecules (AIM) analysis: AIM analysis is based on Bader’s Quantum Theory of Atoms in Molecules (QTAIM), which examines the topology of the electron density ρ(r) to characterize chemical bonding and intermolecular interactions. In this framework, bond critical points (BCPs) and their associated electron density (ρ), Laplacian of electron density (∇^2^ρ), and energy density descriptors provide quantitative insights into bond strength and nature (covalent vs. non-covalent). In our study, AIM analysis was applied to the carbamate intermediates to quantify the strength of N–C bonds and assess the electronic features at reaction sites, complementing the interaction region indicator (IRI) in evaluating steric and electronic effects during CO_2_ desorption.

5) Interaction region indicator (IRI): The IRI is a powerful tool for visualizing and analyzing weak interactions. It effectively reveals regions of steric repulsion and non-covalent attraction. In our study, IRI analysis, coupled with AIM analysis, was employed to evaluate the strength of N-C bonds in the carbamate intermediates and the steric environment around the reaction sites, providing insights into the energy consumption during desorption.

**Text S3. Supplementary experimental methods**

*Particle-size distribution (PSD) by laser diffraction*: After absorption, the solid product was collected by filtration, freeze-dried, and further vacuum-dried at 50 ℃. The particle-size distribution (PSD) of the dried solid was measured in triplicate using a HELOS-OASIS laser diffractometer (Sympatec, Germany). Because drying can alter slurry microstructure, PSD is reported as evidence of handleability (filterable, micrometer-scale particles) rather than as an in-situ nucleation metric. Reported statistics include D10, D50, D90 and span.

*Thermogravimetric analysis coupled with differential scanning calorimetry (TGA-DSC)*: Thermal behavior of the dried solid product was measured by TGA–DSC under N_2_ atmosphere at a 5 ℃/min heating rate. The resulting mass-loss and heat-flow profiles were used to determine decomposition onsets and endothermic events.

*Potentiodynamic polarization (Tafel) corrosion tests*: Corrosion electrochemical measurements were conducted using the CHI-760 electrochemical workstation in a three-electrode system. A 1 cm × 1 cm × 0.5 cm carbon steel specimen and a 1 cm × 1 cm × 0.1 cm platinum plate acted as the working electrode and the counter electrode, respectively. The Ag/AgCl electrode was used as the reference electrode. The experiment was conducted in an 80 mL sealed glass electrolytic cell, with 25 mL of test liquid weighed for each run. The following test liquids were selected to study the corrosion behavior of carbon steel: MEA, 26DMPZ, MEA-CO_2_, 26DMPZ-CO_2_. Before the corrosion experiments, carbon steels were polished using 1000 grit SiC paper. They were then ultrasonically cleaned in acetone followed by ethanol, rinsed with deionized water, and dried. The three electrodes were immersed in the test fluid to establish a steady-state open-circuit potential. Taffel measurements started from a cathodic potential of –300 mV to an anodic potential of +300 mV (versus open circuit potential) at a 0.5 mV/s scan rate.

**2. Results**

**Table S1** Basis set size effects on CO_2_-capture energetics of 26DMPZ: activation free energies (ΔG‡) and reaction free energies (ΔG) at 313.15 K (kcal/mol) computed with M06-2X-D3(0).

| Pathway | Quantity | Basis set | | |
| --- | --- | --- | --- | --- |
|  |  | def2-TZVP | def2-TZVPP | def2-QZVP |
| 26DMPZa | ΔG‡ (kcal/mol) | 2.14 | 2.16 | 2.21 |
|  | ΔG (kcal/mol) | –3.75 | –3.75 | –3.63 |
| 26DMPZb | ΔG‡ (kcal/mol) | 2.16 | 2.17 | 2.23 |
|  | ΔG (kcal/mol) | –5.87 | –5.85 | –5.75 |

**Table S2** Comparison of computed ΔG‡ and ΔG (kcal/mol) for the zwitterion formation across multiple levels of theory.

| Method |  | PZ | NMPZ | NEPZ | NIPZ | 2MPZ | | 26DMPZ | | N2AEPZ | N2HEPZ |
| --- | --- | --- | --- | --- | --- | --- | --- | --- | --- | --- | --- |
|  |  |  |  |  |  | a | b | a | b |  |  |
| revDSD-PBEP86-D3(BJ)/  def2-TZVPP | ΔG‡ | 2.84 | 2.34 | 2.61 | 2.36 | 2.57 | 2.37 | 2.20 | 2.14 | 2.76 | 2.56 |
|  | ΔG | –1.12 | –1.91 | –3.08 | –3.61 | –3.34 | –3.84 | –1.79 | –3.68 | –3.24 | –3.07 |
| M06-2X-D3(0)/  def2-TZVP | ΔG‡ | 2.77 | 2.26 | 2.55 | 2.32 | 2.52 | 2.42 | 2.14 | 2.16 | 2.68 | 2.50 |
|  | ΔG | –3.21 | –4.01 | –5.07 | –5.57 | –5.22 | –5.78 | –3.75 | –5.87 | –5.17 | –4.98 |
| M06-2X/  def2-TZVP | ΔG‡ | 2.76 | 2.25 | 2.53 | 2.30 | 2.52 | 2.41 | 2.14 | 2.15 | 2.66 | 2.48 |
|  | ΔG | –3.22 | –4.03 | –5.09 | –5.59 | –5.24 | –5.81 | –3.75 | –5.91 | –5.19 | –5.01 |

**Table S3** Particle-size distribution (PSD) of dried 26DMPZ dicarbamate (laser diffraction).

| Run | D10 (μm) | D50 (μm) | D90 (μm) | Span=(D90-D10)/D50 |
| --- | --- | --- | --- | --- |
| 1 | 7.72 | 64.51 | 178.98 | 2.65 |
| 2 | 15.59 | 94.86 | 195.52 | 1.90 |
| 3 | 17.18 | 91.71 | 184.35 | 1.82 |
| Mean ± SD | 13.5 ± 4.2 | 83.7 ± 13.9 | 186.3 ± 8.6 | 2.1 ± 0.4 |

**Table S4** Glossary of abbreviations and key terms.

| Abbreviations | Definition |
| --- | --- |
| 26DMPZ | 2,6-dimethylpiperazine |
| 26DMPZdc^2–^ | the dicarbamate dianion of 26DMPZ |
| 26DMPZH^+^ | the protonated form of 26DMPZ |
| 26DMPZmc^–^ | the mono-substituted carbamate anion of 26DMPZ |
| 2MPZ | 2-methylpiperazine |
| AEEA | 2-((2-aminoethyl)amino)ethanol |
| AIM | Atoms-in-molecules |
| AMP | 2-amino-2-methyl-1-propanol |
| CCUS | Carbon capture, utilization, and storage |
| DFT | Density functional theory |
| DMF | N,N-dimethylformamide |
| DMMEA | Dimethyl monoethanolamine |
| DMSO | Dimethyl sulfoxide |
| ESP | Electrostatic potential |
| ESP_H | ESP at hydrogen nuclei |
| FTIR | Fourier-transform infrared |
| HRMS | High-resolution mass spectrometry |
| I_corr_ | the corrosion currents |
| IRC | Intrinsic reaction coordinate |
| IRI | Interaction-region indicator |
| MDEA | N-methyldiethanolamine |
| MEA | Monoethanolamine |
| MMEA | Methyl monoethanolamine |
| N2AEPZ | N-(2-Aminoethyl)piperazine |
| N2HEPZ | N-(2-Hydroxyethyl)piperazine |
| NEPZ | N-Ethylpiperazine |
| NIPZ | N-Isopropylpiperazine |
| NMPZ | N-methylpiperazine |
| NMR | Nuclear magnetic resonance |
| PMDETA | Pentamethyldiethylenetriamine |
| PSD | Particle size distribution |
| PZ | Piperazine |
| RDG | Reduced density gradient |
| SMD | Solvation model based on solute electron density |
| TEDA | Triethylenediamine |
| TETA | Triethylenetetramine |
| TGA-DSC | Thermogravimetric analysis coupled with differential scanning calorimetry |
| vdW | Van der Waals |
| VOCs | Volatile organic compounds |
| α_e_ | Equilibrium solubility |
| ΔG | Gibbs free energy change |
| ΔG‡ | Activation barrier |
| ΔG_solv_ | Solvation free energy |
| ΔH_abs_ | Absorption enthalpy |


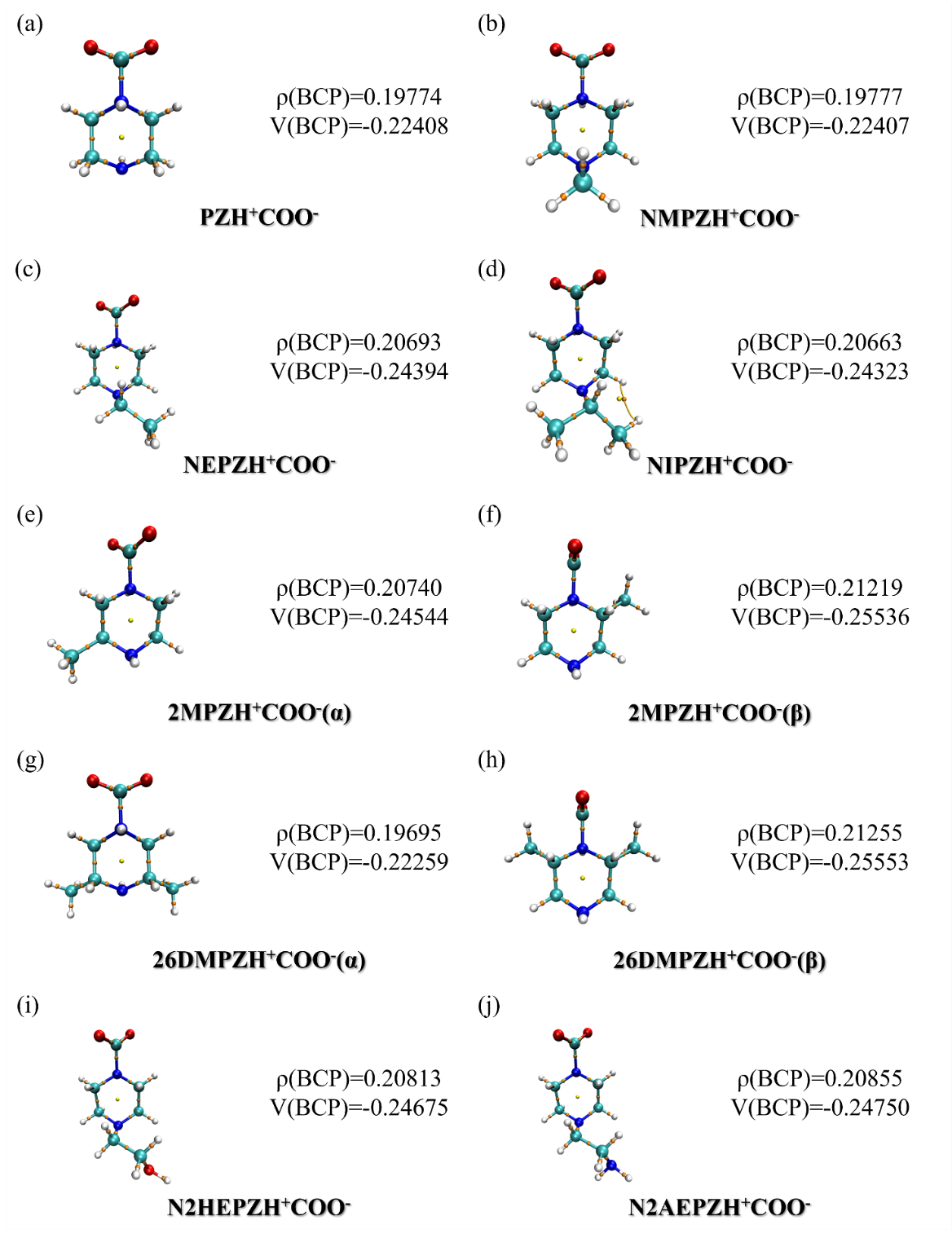


**Figure S1.** ρ(BCP) and V(BCP) of the interaction bond critical points of PZ derivatives’ zwitterion.


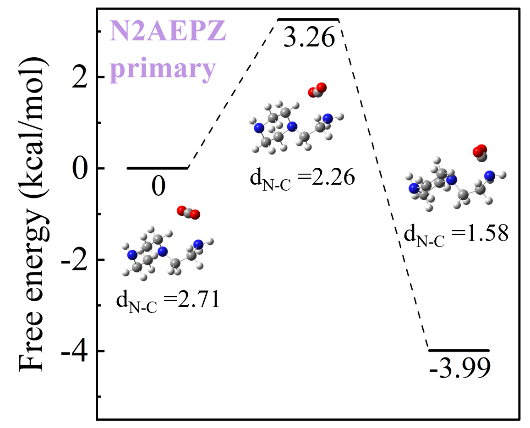


**Figure S2.** Energy changes for zwitterion formation of N2AEPZ’s primary amine group.


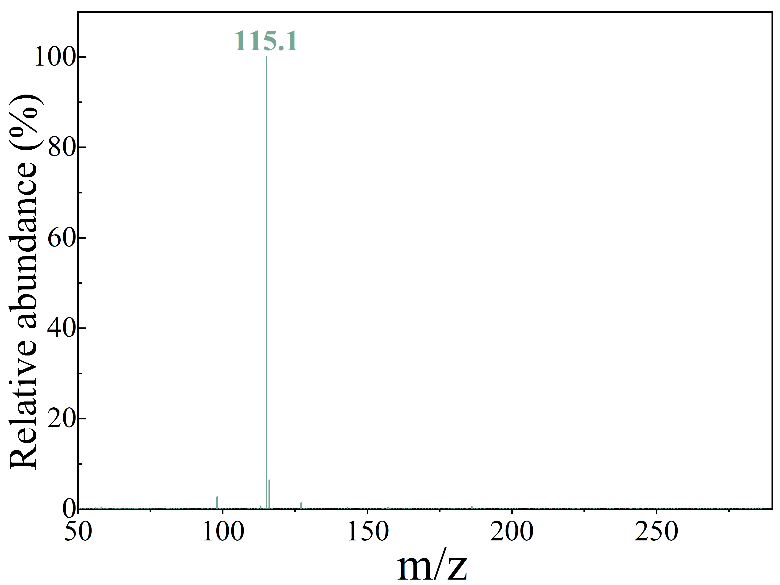


**Figure S3.** Positive-mode ESI mass spectrum of the solid product


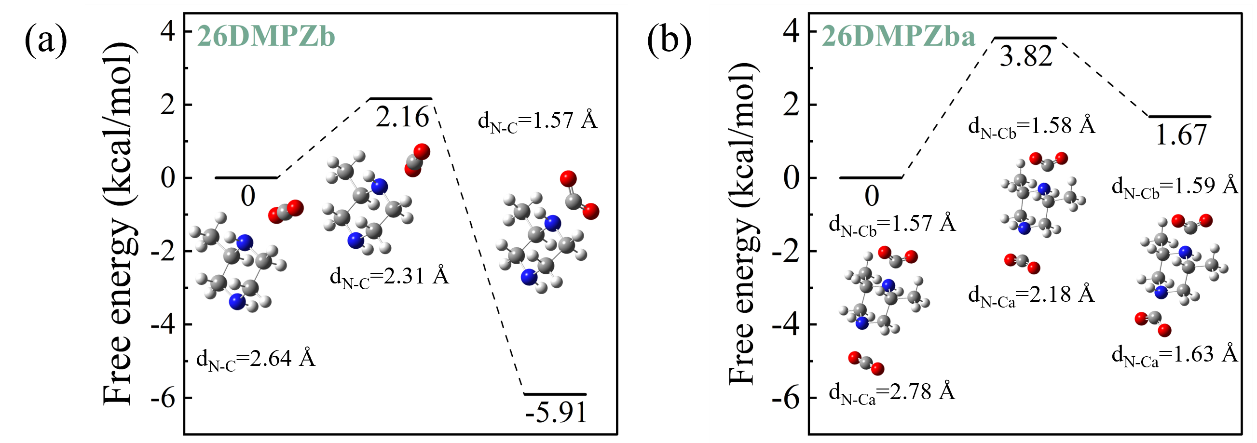


**Figure S4.** ΔG‡ and ΔG for the formation of: (a) monocarbamate from 26MDPZ and CO_2_; (b) dicarbamate intermediate from the monocarbamate intermediate.


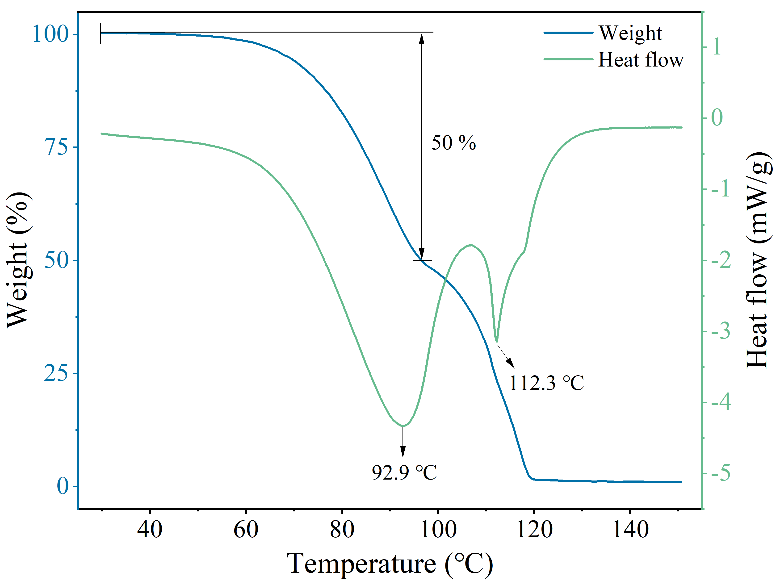


**Figure S5.** TGA-DSC profiles of the solid product: thermal behavior analysis under N_2_ atmosphere at a heating rate of 5 ℃/min.


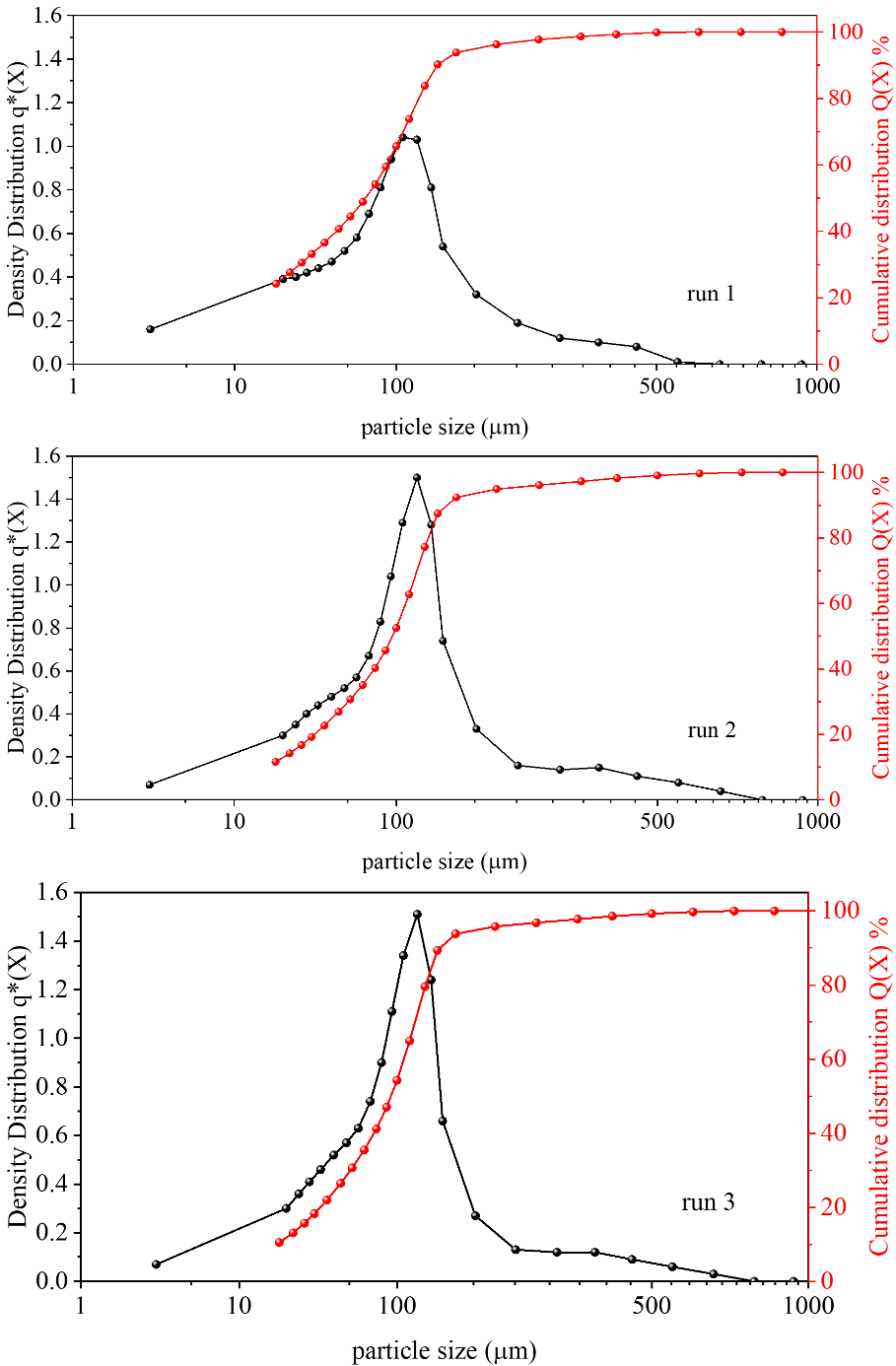


**Figure S6.** Comparison of density and cumulative distributions of the solid product particle sizes across different runs.


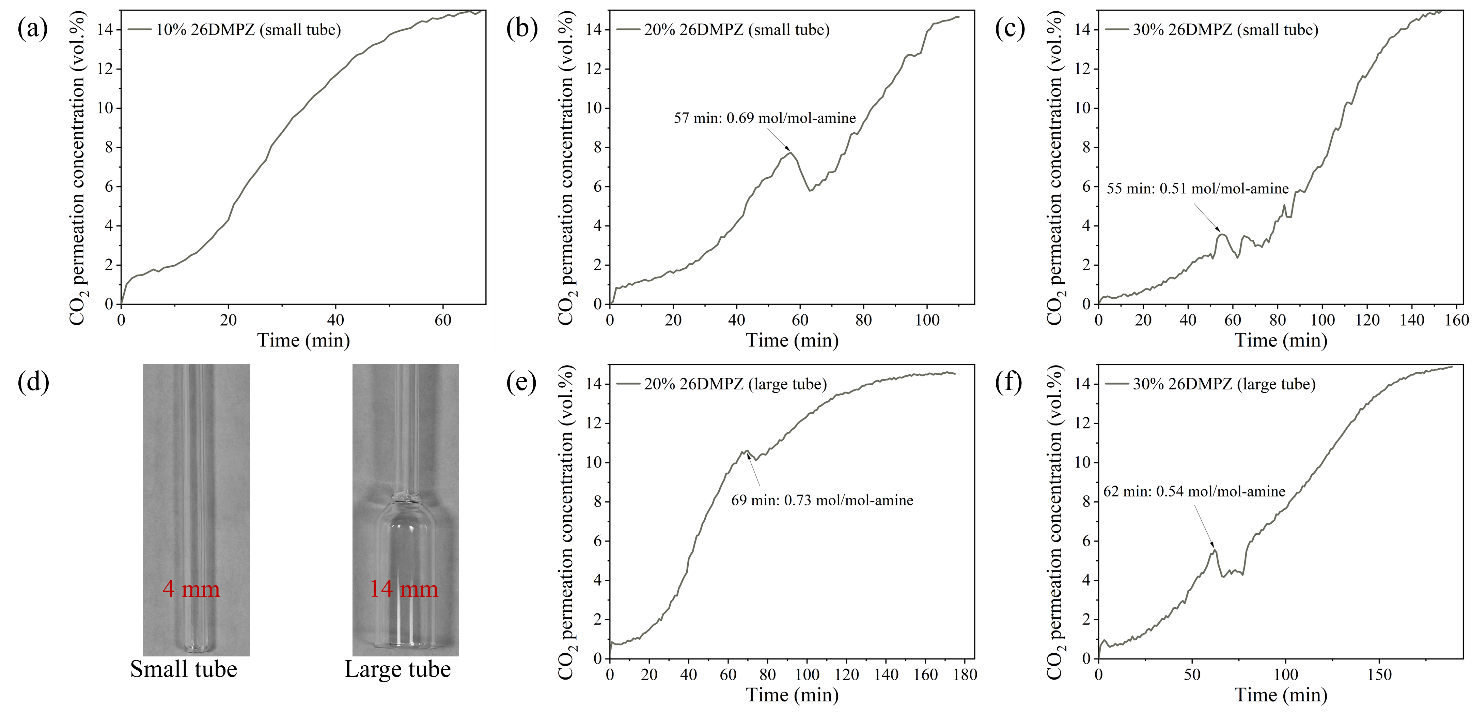


**Figure S7.** CO_2_ breakthrough profiles and precipitation onset for 26DMPZ solutions under varying concentrations and reactor geometries.


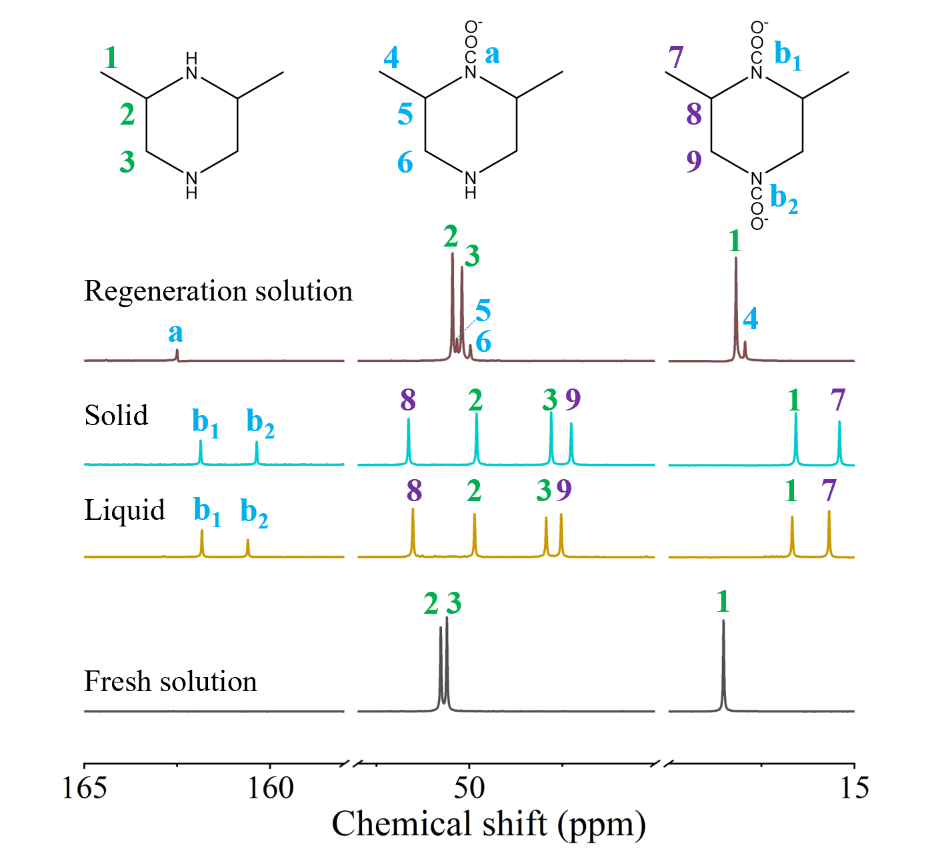


**Figure S8.** ^13^C NMR spectra of 26DMPZ: fresh solution, liquid phase, solid phase and regeneration solution.


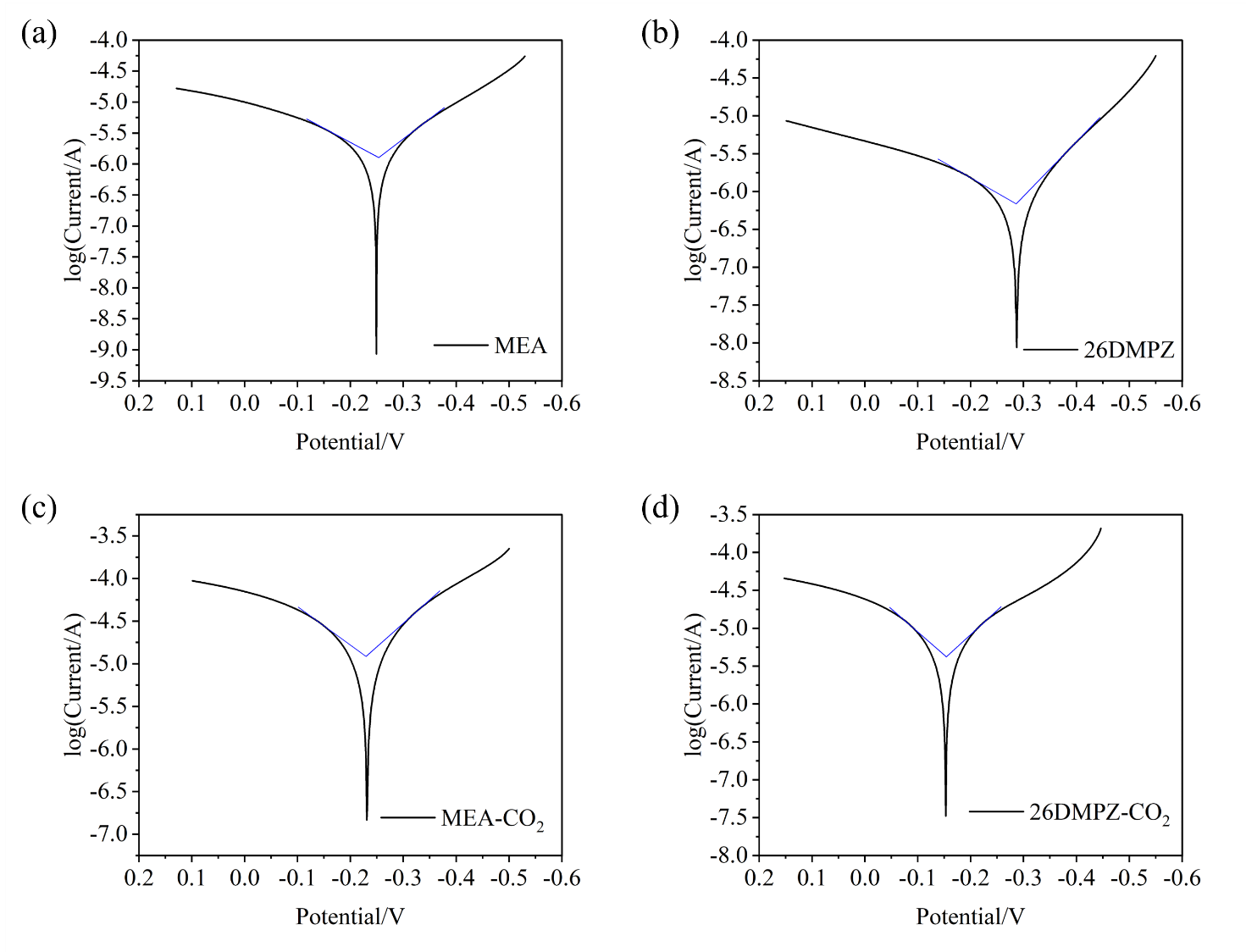


**Figure S9.** Tafel plots for carbon steel working electrode in test fluids of (a) MEA solution, (b) 26DMPZ solution, (c) CO_2_-loaded MEA solution, and (d) CO_2_-loaded 26DMPZ solution.


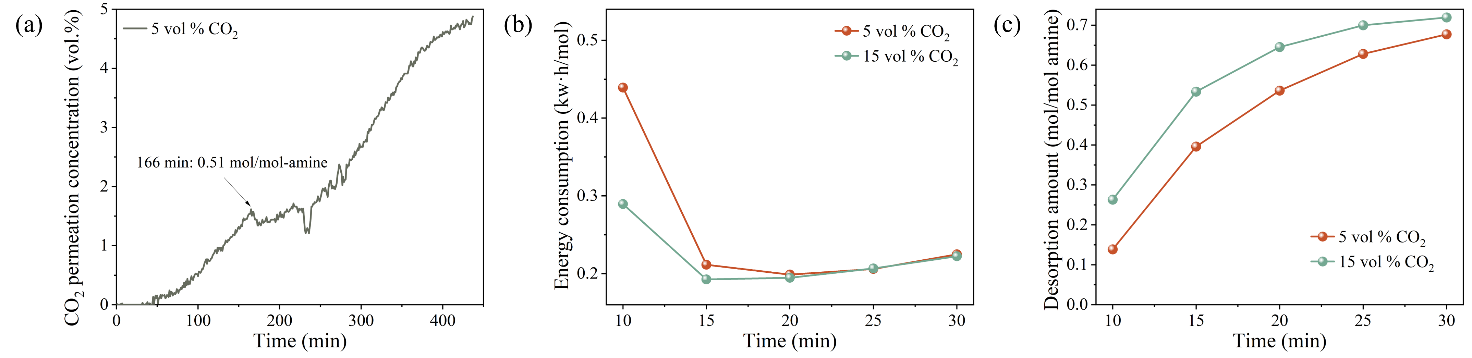


**Figure S10.** Experimental evaluation of absorption and desorption performance of 26DMPZ under 5 vol% CO_2_ atmosphere: (a) CO_2_ breakthrough concentration during the absorption process; (b) time-dependent energy consumption during desorption; (c) variation in CO_2_ desorption amount over 30min.


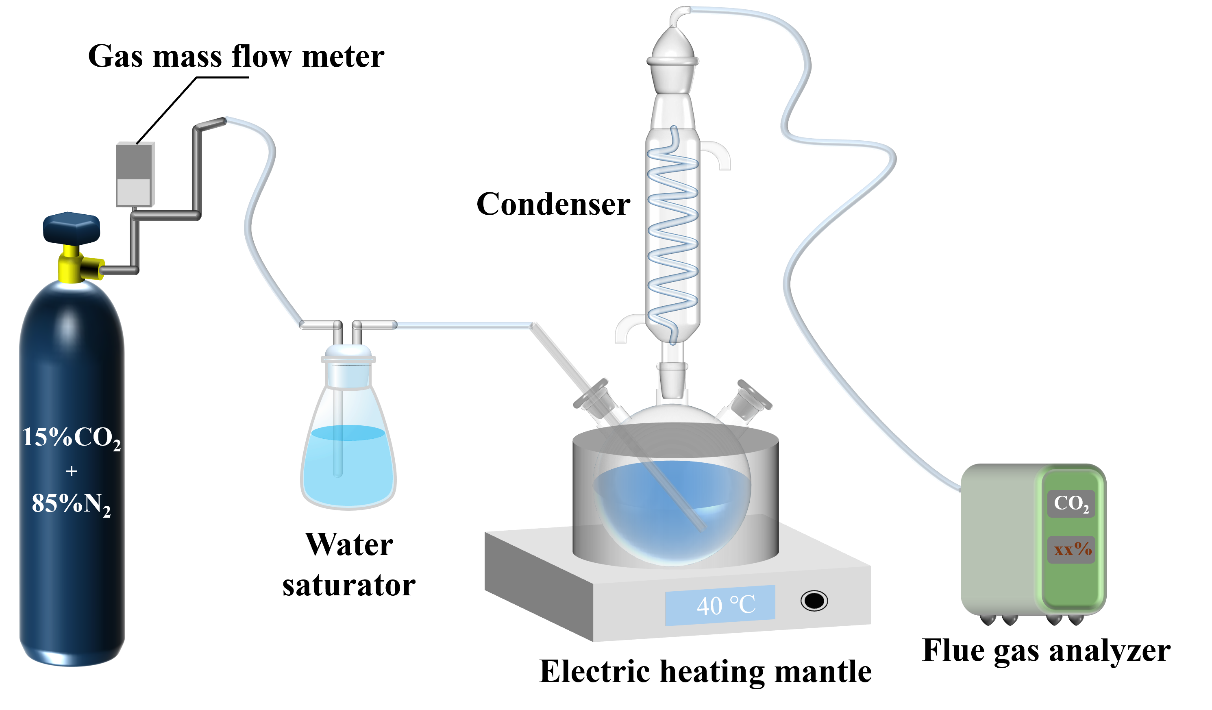


**Figure S11.** Laboratory CO_2_ absorption system.


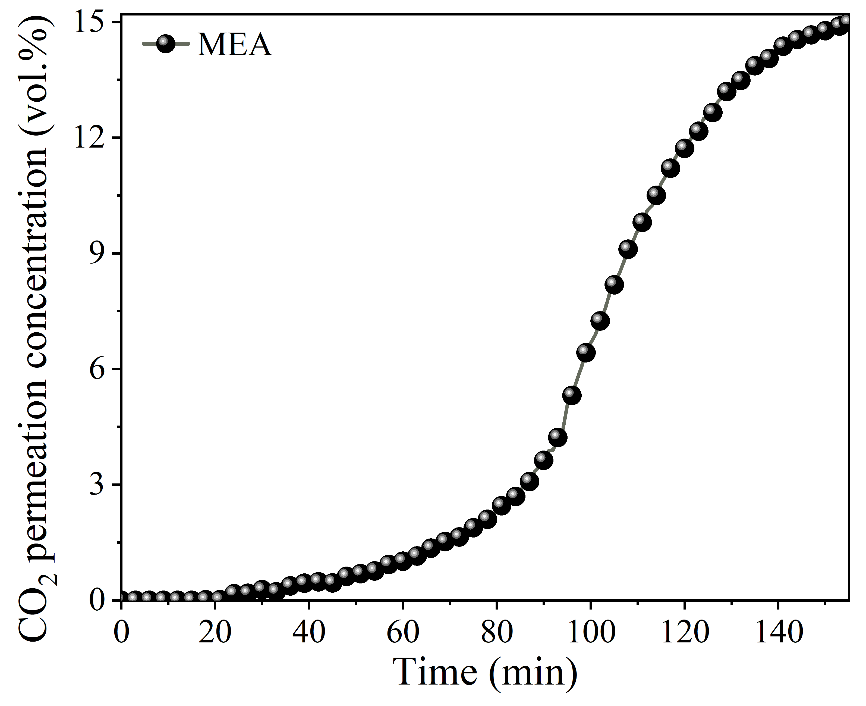


**Figure S12.** CO_2_ permeation concentration over time of 30 wt.% MEA solution.


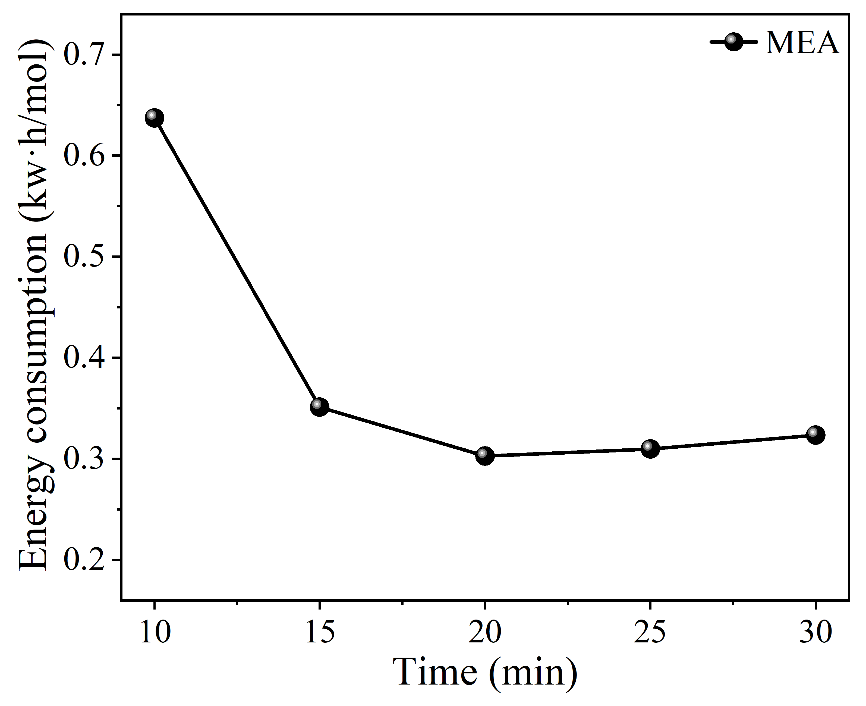


**Figure S13.** Time-dependent energy consumption during desorption of MEA.


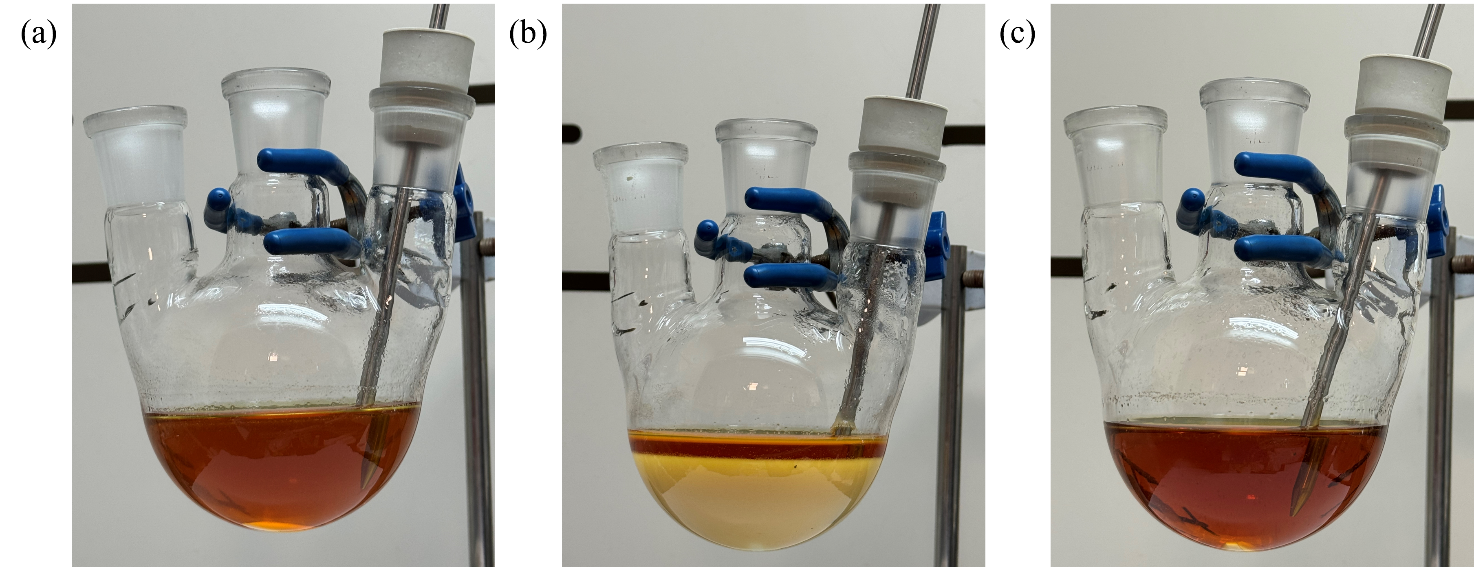


**Figure S14.** (a) the fresh 26DMPZ solution, (b) the solid-liquid mixture after CO_2_ absorption, and (c) the regeneration solution.

**References**

[1] J. Ho, A. Klamt, M. L. Coote, *J. Phys. Chem. A* **2010**, *114* (51), 13442-13444.
